# Characterization and Dynamics of Repeatomes in Closely Related Species of *Hieracium* (Asteraceae) and Their Synthetic and Apomictic Hybrids

**DOI:** 10.3389/fpls.2020.591053

**Published:** 2020-11-02

**Authors:** Danijela Zagorski, Matthias Hartmann, Yann J. K. Bertrand, Ladislava Paštová, Renata Slavíková, Jiřina Josefiová, Judith Fehrer

**Affiliations:** Institute of Botany, Czech Academy of Sciences, Průhonice, Czechia

**Keywords:** apomixis, hybridization, polyploidization, RepeatExplorer, next-generation sequencing, repeatome, hawkweed

## Abstract

The repetitive content of the plant genome (repeatome) often represents its largest fraction and is frequently correlated with its size. Transposable elements (TEs), the main component of the repeatome, are an important driver in the genome diversification due to their fast-evolving nature. Hybridization and polyploidization events are hypothesized to induce massive bursts of TEs resulting, among other effects, in an increase of copy number and genome size. Little is known about the repeatome dynamics following hybridization and polyploidization in plants that reproduce by apomixis (asexual reproduction via seeds). To address this, we analyzed the repeatomes of two diploid parental species, *Hieracium intybaceum* and *H. prenanthoides* (sexual), their diploid F1 synthetic and their natural triploid hybrids (*H. pallidiflorum* and *H. picroides*, apomictic). Using low-coverage next-generation sequencing (NGS) and a graph-based clustering approach, we detected high overall similarity across all major repeatome categories between the parental species, despite their large phylogenetic distance. Medium and highly abundant repetitive elements comprise ∼70% of *Hieracium* genomes; most prevalent were Ty3/Gypsy chromovirus Tekay and Ty1/Copia Maximus-SIRE elements. No TE bursts were detected, neither in synthetic nor in natural hybrids, as TE abundance generally followed theoretical expectations based on parental genome dosage. Slight over- and under-representation of TE cluster abundances reflected individual differences in genome size. However, in comparative analyses, apomicts displayed an overabundance of pararetrovirus clusters not observed in synthetic hybrids. Substantial deviations were detected in rDNAs and satellite repeats, but these patterns were sample specific. rDNA and satellite repeats (three of them were newly developed as cytogenetic markers) were localized on chromosomes by fluorescence *in situ* hybridization (FISH). In a few cases, low-abundant repeats (5S rDNA and certain satellites) showed some discrepancy between NGS data and FISH results, which is due partly to the bias of low-coverage sequencing and partly to low amounts of the satellite repeats or their sequence divergence. Overall, satellite DNA (including rDNA) was markedly affected by hybridization, but independent of the ploidy or reproductive mode of the progeny, whereas bursts of TEs did not play an important role in the evolutionary history of *Hieracium*.

## Introduction

Repetitive elements, collectively known as the ‘repeatome’ (Maumus and Quesneville, 2014), comprise DNA fragments that are present in multiple copies throughout the genome. Due to their fast-evolving nature and tendency toward accumulation, they represent one of the most important factors contributing to the remarkable variation of genome size in plants (Bennetzen et al., 2005). Based on their genome organization, repetitive elements fall into two main categories: tandem repeats and transposable elements (TEs).

Tandem repeats are composed of multiple copies of the same DNA sequence (monomers) arrayed in a head-to-tail fashion. They are usually classified based on the length of the monomer into microsatellite (up to 10 bp), minisatellite (10–60 bp), and satellite repeats (from tens of base pairs up to several kilobases (Stupar et al., 2002; Ávila Robledillo et al., 2018). Their amount, chromosomal distribution, and homology may be genus-, species-, genome- or chromosome-specific, which consequently make them useful markers for inheritance studies of interspecific hybrids (Hemleben et al., 2007).

TEs are DNA sequences that can copy and insert themselves to different locations within a genome. TEs can be classified according to their mode of transposition: Class I (retrotransposons) that replicate and transpose via an RNA intermediate, and Class II (DNA transposons) that directly excise themselves and insert into a new location without any intermediate. Each class is divided into further subcategories based on the presence and structure of protein domains and different non-coding sequences (Wicker et al., 2007). In plants, TE content is highly variable, e.g., from 10% in *Arabidopsis thaliana*, to over 85% in the maize genome (Dubin et al., 2018).

TEs play many important roles such as shaping the genome architecture (reviewed in Galindo-González et al., 2017). Furthermore, TEs can alter gene expression if inserted into genes or promoter regions. TEs are controlled by various silencing mechanisms (Okamoto and Hirochika, 2001), therefore factors decreasing silencing efficacy can trigger TE activity.

McClintock (1984) hypothesized that hybridization in plants may represent a ‘genomic shock’ inducing bursts of TEs, which may lead to massive genome reorganization in newly formed hybrids. Similar effects of TE activation and transposition have been suggested for polyploidization (Comai, 2000). In line with this hypothesis, several investigations into the effects of hybridization and polyploidization on TEs (mostly in synthetic or recent natural hybrids and allopolyploids) showed that some TEs responded with increased transcriptional activity (e.g., Kashkush et al., 2002; Madlung et al., 2005), methylation changes (Xu et al., 2009; Kraitshtein et al., 2010; Yaakov and Kashkush, 2010), and induction of changes in genome structure (Madlung et al., 2005). However, only few studies have detected bursts of TEs that resulted in an increase of copy number (Petit et al., 2010; Ben-David et al., 2013; An et al., 2014; Hake et al., 2018) and transposon mobility (Shan et al., 2005) in response to hybridization and polyploidization events, whereas a number of studies showed no or only limited TE transposition (e.g., Madlung et al., 2005; Beaulieu et al., 2009; Parisod et al., 2009; Mestiri et al., 2010; Sarilar et al., 2013; see also reviews of Parisod and Senerchia, 2012 and Vicient and Casacuberta, 2017). Some studies reported the increased amplification of certain TEs in natural hybridogenous and polyploid species compared to their diploid ancestors (e.g., Sarilar et al., 2011; Piednoël et al., 2015). Thus, TE response to hybridization and polyploidization differs between genomes and types of TEs, indicating that TE bursts are not a general consequence of these processes (Shan et al., 2005; Parisod and Senerchia, 2012; Belyayev, 2014; Vicient and Casacuberta, 2017). Also, the influence of other factors that trigger TE activity such as environmental stress (Casacuberta and González, 2013) in the study of old hybrids and polyploids cannot be excluded.

The mode of reproduction may constitute one of the major factors affecting plant genome size and TE content. Theoretical predictions about the direction of TE content change (and consequently the increase or decrease of genome size) in selfers and asexuals in comparison to their sexual relatives, remain unclear (Glémin and Galtier, 2012). In the simplest scenario, sexual reproduction represents the most favorable means for TEs to spread horizontally to all lineages within the population (Hickey, 1982). Despite being considered as selfish elements that have mostly adverse effects on the host’s fitness, TEs can develop maximum transposition rates in sexual populations (Charlesworth and Langley, 1986). Conversely, in asexual populations, the propagation of TEs is limited to vertical (within-lineage) transmission. As asexuals are usually derived from sexual progenitors, they inherit their parental TE load, including active elements. In order to survive the detrimental proliferation of TEs, asexuals should purge and inactivate their TE load by means of self-regulatory mechanisms. Ultimately, over longer evolutionary time, those asexual lineages with a more efficiently inactivated TE load would be selected over lineages with higher TE proliferation rates. In this case, it is expected that asexuals would have smaller TE contents and genome sizes than sexuals (Dolgin and Charlesworth, 2006; Ågren et al., 2015). On the other hand, as the purging can take a very long time, in small asexual populations, due to genetic drift, a Muller’s ratchet-like process will drive the accumulation of TEs (Dolgin and Charlesworth, 2006; Glémin and Galtier, 2012). Under this scenario, it is expected that asexuals would have a higher TE content and genome size than their sexual relatives. Furthermore, asexually reproducing plants often emerge through the process of hybridization of (predominantly) sexual species, which is almost always followed by polyploidization. These events may be accompanied by massive genome re-patterning, including bursts of TEs, which additionally complicates the study of the influence of the reproductive mode on repeatome dynamics in asexual plants. It is therefore of invaluable interest to disentangle the consequences of hybridization and/or polyploidization from the consequences of the transition to the asexual mode of reproduction.

The theoretical predictions are mostly based on computer simulations, and empirical studies of repeatomes in asexually reproducing plants are scarce (e.g., Docking et al., 2006; Ågren et al., 2015; Ferreira de Carvalho et al., 2016). Until recently, technologies suitable for studying TEs in plants were not available and repeatome studies were mostly focused on a small number of model plant species. The advent of next-generation sequencing (NGS) and bioinformatics tools such as the graph-based clustering approach (Novák et al., 2010, 2013) have enabled the comprehensive characterization of repeatomes of non-model plant species at low cost and without a need for an assembled genome.

The genus *Hieracium* L. (Asteraceae) represents a model system for the study of apomixis, which is asexual reproduction via seeds. Concerted events of hybridization, polyploidization, and shifts to apomixis have played a major role in *Hieracium* evolution (Fehrer et al., 2009). This genus consists of ca. 25 sexual diploids (*x* = 9) and 500–5000 (Majeský et al., 2017) polyploid taxa (mostly triploid, 2*n* = 3*x* = 27, and tetraploid, 2*n* = 4*x* = 36); the latter reproduce almost exclusively apomictically (Mráz and Zdvořák, 2019). Diploid species are well differentiated morphologically, and together with several apomictic polyploid taxa they form so-called ‘basic’ species (altogether ca. 45 species) and are considered as main units of species evolution in the genus *Hieracium* (Chrtek et al., 2009). The rest of polyploid taxa are considered ‘intermediate’ species as they share morphological characters of two or more basic species and are supposed to be of hybridogenous origin. The division on ‘basic’ and ‘intermediate’ species is still largely based on morphology.

In a recent study, Chrtek et al. (2020) used complementary phylogenetic and cytogenetic approaches to study one such system consisting of two parental ‘basic’ species, *H. intybaceum* (Int) and *H. prenanthoides* (Pre), and two ‘intermediate’ triploid species, *H. pallidiflorum* (Pal) and *H. picroides* (Pic). The authors demonstrated that the two ‘intermediates’ indeed originated through hybridization of the parental pair as suggested by their morphology. The genome dosage of *H. pallidiflorum* (morphologically closer to *H. intybaceum*) is 2 Int : 1 Pre, and that of *H. picroides* (morphologically closer to *H. prenanthoides*) is 1 Int : 2 Pre. The two apomictic lineages have each originated independently multiple times (polytopic speciation). In addition to these natural polyploid hybrids, experimental crosses between the parental species successfully produced synthetic F1 diploid sexual hybrids (genome dosage 1 Int : 1 Pre) with intermediate morphology. Therefore, this system–consisting of the diploid parental species, old apomictic allopolyploids, and synthetic F1 sexual diploid hybrids–provides an excellent model to investigate and compare the short- and long-term consequences of hybridization and polyploidization events, followed by the shift to apomixis.

Chrtek et al. (2020) carried out basic repeatome comparisons of the parental species *H. intybaceum* and *H. prenanthoides*, and genomic *in situ* hybridization (GISH) on their hybrids. The analyses revealed a surprising repeatome similarity of the parental species, that is not reflected by their large phylogenetic divergence based on several molecular markers (Fehrer et al., 2007, 2009; Krak et al., 2013). The same study showed small, but stable differences in genome size between the parental species. Genomes of allopolyploid *H. pallidiflorum* samples were slightly larger than the sum of the two parental genomes (theoretical expectation) while the genomes of allopolyploid *H. picroides* samples showed more diversity, with both higher or lower sizes compared to the theoretical expectation.

In the present contribution, we perform a detailed repeatome analysis of the plant model system introduced by Chrtek et al. (2020), using the graph-based clustering approach (Novák et al., 2010, 2013). We aim to: 1) characterize the repeatomes of the parental *Hieracium* species and their natural apomictic and synthetic F1 hybrids, 2) compare diploid synthetic hybrids and allopolyploid apomicts to their parental species to detect changes in the repeatome following hybridization and polyploidization events, and 3) compare the repeatomes of synthetic hybrids and allopolyploid apomicts in order to discover repeatome patterns or compositions that would be apomict-specific. As we discovered a significant deviation in the amount of rDNA and satellite repeats in apomicts and synthetic hybrids, we localized these loci on the chromosomes by fluorescence *in situ* hybridization (FISH).

## Materials and Methods

### Plant Material, Sampling, and Genome Size Estimation

In total, eleven accessions have been included in this study. We sampled two individuals for each of the diploid parental species (*H. intybaceum* [Int], *H. prenanthoides* [Pre]) and their natural triploid hybrids (*H. pallidiflorum* [Pal], *H. picroides* [Pic]) and three for the synthetic F1 diploid sexual hybrids (Hyb). Samples of the triploids were chosen from a larger set of polytopic populations, based on their intraspecific differences in genome size and maternal origins (Pic) or different geographic origins (Pal). Pic was represented with two accessions that showed the highest (accession no.: H1613, ID: PicF) and the lowest genome size (accession no.: H1615, ID: PicB). Their genome sizes were slightly outside of the range of genome sizes that would be theoretically expected based on summation of the genome sizes of parental species. Samples of Pal (accession no.: H1609 [ID: PalA] and accession no.:H1614 [ID: PalF]) had slightly higher genome size than theoretically expected (data from Chrtek et al., 2020). Synthetic hybrids (accession no.: 17038_2, 17038_3 and 17038_4 [IDs: Hyb2, Hyb3 and Hyb4]) were obtained from the same homoploid cross between two of the parental accessions included in this study (accession no.: int_1531/8 [IntA] and pre_6/8/5 [PreC]). Details about the accessions’ origins and genome sizes are provided in Table 1.

**TABLE 1:** List of samples, genome size, and initial NGS datasets.

Species, accession labels	ID	Locality^a^	Ploidy level	Genome size 2C (pg)	No. of raw NGS pair-end reads
***H. intybaceum***					
int_1531/8	IntA	Austria, Tirol, Arlberg Massif: Arlbergpass	2x	7.540 ^a^	45,992,578
					27,990,512 ^d^
int_6/14/25	IntC	France, Savoie: Col du Petit Saint-Bernard	2x	7.590^a^	37,998,498
					35,974,348^d^
***H. prenanthoides***					
pre_6/5/5	PreA	Italy, Piedmont: Claviere	2x	7.220^a^	52,429,354
					45,062,158 ^d^
pre_6/8/5	PreC	Italy, Piedmont: Claviere	2x	7.180^*b*^	26,260,212
***H. pallidiflorum* (2 Int × 1 Pre)**					
H1609	PalA	Austria, Bundesland Salzburg: Muhr	3x	11.334^*a*^	24,970,582
H1614	PalF	France, Savoie: Col du Petit Saint-Bernard	3x	11.584^a^	28,335,496
***H. picroides* (1 Int × 2 Pre)**					
H1613	PicF	France, Hautes-Alpes: Col du Lautaret	3x	11.412^a^	30,698,202
H1615	PicB	France, Savoie: Col du Petit Saint-Bernard	3x	10.659^a^	26,850,644
**Synthetic F1 hybrids *H. intybaceum* × *H. prenanthoides* (IntA × PreC)**					
17038_2	Hyb2	experimental garden, Průhonice, Czechia	2x	7.360^c^	26,922,000
17038_3	Hyb3	experimental garden, Průhonice, Czechia	2x	7.360^c^	24,944,800
17038_4	Hyb4	experimental garden, Průhonice, Czechia	2x	7.341^a^	26,225,800

*^*a*^For details of accession origin, voucher information and genome sizes, see Chrtek et al. (2020).^*b*^mean value of all genome size measurements for diploid *H. prenanthoides* from Chrtek et al. (2009), Chrtek et al. (2020).^*c*^values are calculated based on the average of genome sizes of parental accessions.^*d*^for samples IntA, IntC and PreA two independent libraries were sequenced.*

### DNA Isolation, NGS Sequencing, and Reads’ Pre-processing

DNA was extracted from fresh or silica-gel dried leaf tissue using the DNeasy Plant Mini Kit (Qiagen, Hilden, Germany). Library preparation and low-coverage Illumina NGS were performed at GATC Biotech (Konstanz, Germany) / Eurofins Genomics (Ebersberg, Germany) using a standardized protocol that produced 150 bp paired-end reads with an insert size of ∼450 bp. For selected parental accessions (IntA, IntC and PreA), two independent libraries were prepared and sequenced in order to test for potential bias at the level of library preparation. The sequencing resulted in datasets of 25–54 million individual pair-end reads (i.e., 12.5–27 million of pairs) per library (Table 1). The raw Illumina datasets have been submitted to the European Nucleotide Archive (ENA) under study no. PRJEB35856. Quality filtering was performed using the pre-processing tool included in the public Galaxy server running the RepeatExplorer pipeline,^1^ with a Phred quality score 10 over at least 95% of the bases in a sequence, with no ambiguous bases (Ns) allowed. Only proper pairs of reads of the same length (150 bp) were retained. Reads were scanned for overlap, and only non-overlapping pairs of reads were kept for further analysis in order to increase the representation of the genome. In the case of datasets used for comparative analyses (see below), prior filtering of plastid and mitochondrial reads was included because of the high variability in the amount of chloroplast sequences among samples (3–13% of the total reads) that may distort the quantification of TEs. The filtering was done with the script ‘bbsplit.sh’ from BBTools v.37.44^2^ using the most closely related genomes currently available, i.e., the chloroplast genome of *Lactuca sativa* (DQ383816.1) and the mitochondrial genome of *Helianthus annuus* (MG770607.2).

### Graph-Based Clustering Approach

The repeatomes of all accessions were analyzed using the graph-based clustering approach, as described in Novák et al. (2010, 2013) employing the RepeatExplorer (RE) pipeline implemented within the Galaxy server. This approach allows *de novo* identification of repetitive elements from unassembled reads obtained from low-coverage genome sequencing. The pipeline performs all-to-all BLAST comparisons of the input reads (using a threshold of 90% or higher similarity over at least 55% of the read length), which is then used to construct a graph wherein reads are represented as vertices and their sequence similarities as edges. The graph is further partitioned into highly connected communities of vertices (called ‘clusters’), which tends to cluster reads from the same family of repetitive elements. The clusters of reads are quantified, annotated and reads within clusters are assembled into contigs. In addition to clusters, the pipeline also produces superclusters, which represent groups of clusters that share split pair-end reads. Superclusters occur when the reads from the same read pair (which originate from the same insert in the NGS library) end up in two separated clusters during the clustering phase, and therefore such clusters are assumed to originate from a single repeat. Thus, information about shared pair-end reads can be used in annotation of otherwise unannotated clusters, if they are connected to annotated clusters within the supercluster. Following the criteria described in McCann et al. (2018), clusters were considered to belong to the same supercluster, if the ratio of the number of pair-end reads shared between clusters to the sum of the total number of unpaired reads in each cluster was higher than 0.1.

### Testing the Bias Due to Library Preparation and Similarity Between Parental Accessions

Library preparation for NGS can be a source of significant bias (van Dijk et al., 2014), especially when dealing with closely related/highly similar samples. We determined how this bias affects cluster quantification based on different libraries from the same accession. We sequenced two independently prepared libraries for three selected parental accessions (IntA, IntC and PreA; Table 1). The bias was investigated using the functionality of RE comparative clustering, where we performed a set of ‘intra-library’ comparative runs (comparison of two independent subsamples of 2 million reads originating from the *same* library) and ‘inter-library’ comparative runs (comparison of independent subsamples of 2 million reads originating from *different* libraries of the same accession). The read datasets were created using a random sampling tool on the Galaxy server. The variation in cluster sizes in those comparative runs was statistically assessed using a pairwise Wilcoxon test. To this extent, the absolute difference in read proportion across all clusters was used as response. The results of intra-library and inter-library comparisons were further assessed with intra-specific and inter-specific comparative runs, which were prepared in the same way. All statistical analyses in this study were carried out in R (R Core Development Team, 2018).

### Individual Clustering Analysis and Repeat Identification

For annotation purposes, all accessions were first analyzed individually using the maximum number of pair-end reads (Table 2) allowed by the pipeline, given the available computational resources (max. 112 GB of RAM) and depending on the repeat content of the analyzed genome (i.e., the more repetitive a genome, the smaller the number of reads that can be used). Using the highest possible number of reads ensures the best accuracy of the annotation and maximizes the possibility of detecting repeats with lower genomic proportions. Clusters were quantified, and those containing at least 0.01% of the total read input were automatically annotated using the DNA and reference TEs domain database available within RE (Viridiplantae version 2.2). The annotation results were manually checked and corrected. Clusters from plastid and mitochondrial origins were identified and excluded from further analysis. Following McCann et al. (2018), clusters were annotated if at least 5% of their reads produced a BLAST hit to one or more protein domains that belong to the same lineage of transposable elements. Additionally, in order to improve cluster annotation, their contigs were subjected to a RepeatMasker search against the Viridiplantae database (Jurka et al., 2005) and to BLASTn and BLASTX searches against public databases.^3^ Tandem Repeat Finder (Benson, 1999) and the YASS genomic similarity tool^4^ (Noé and Kucherov, 2005) were used for the discovery of potential tandem repeats. Finally, annotated contigs of parental species were used to double-check the annotation of clusters of natural and synthetic hybrids. Annotation was done primarily at the level of superclusters, but also at the level of clusters, and both results were compared.

**TABLE 2:** Number of reads used by RepeatExplorer in individual analyses and main clustering results.

Sample ID and ploidy level	Genome size 1C (Gbp)	No. of analyzed reads^a^	Coverage	Reads in all clusters including small uncharacterized clusters (%)	Reads in clusters above the threshold of 0.01% (%)	Singlets (%)	No. of clusters above the threshold of 0.01%	No. of superclusters above the threshold of 0.01%
IntA (2x)	3.686	4,349,101	0.18 x	82.87	71.54	17.13	246	157
IntC (2x)	3.711	4,270,305	0.17 x	81.69	69.87	18.31	224	145
PreA (2x)	3.531	4,212,792	0.18 x	82.55	71.11	17.45	230	150
PreC (2x)	3.511	4,368,530	0.19 x	81.42	69.48	18.58	228	149
PalA (3x)	5.543	4,278,270	0.12 x	81.39	70.53	18.61	236	149
PalF (3x)	5.664	4,467,725	0.12 x	82.19	70.92	17.81	242	158
PicF (3x)	5.580	3,767,752	0.10 x	80.74	69.96	19.26	209	138
PicB (3x)	5.212	4,338,941	0.12 x	81.31	69.60	18.69	260	173
Hyb2 (2x)	3.600	4,457,370	0.20 x	82.01	71.22	17.99	241	160
Hyb3 (2x)	3.600	4,636,407	0.20 x	81.82	71.06	18.18	251	161
Hyb4 (2x)	3.590	4,465,263	0.19 x	81.83	71.11	18.17	246	157

*^*a*^After exclusion of plastid and mitochondrial clusters.*

### Comparative Clustering Analysis

Besides individual analyses, read datasets of parental species and their natural or synthetic hybrids were subjected to comparative clustering analyses, which precisely detect differences in the abundance of specific sequence variants of repetitive elements. In order to maximize the number of reads per sample, we performed three sets of analyses with a maximum number of three samples in each analysis (Table 3): comparisons between (1) parental species; (2) natural hybrids and their parental species; and (3) synthetic F1 hybrids and their actual parents. As for comparisons in (1) and (2), we created ‘in silico’ parents (IntX and PreX) by pooling reads from all sequenced libraries of both individuals of *H. intybaceum* and *H. prenanthoides*. This approach aims to increase sampling diversity of the parental species since the actual parental genotypes involved in the origins of Pic and Pal accessions are unknown. Read datasets were created by random subsampling and ensuring equal coverage of all samples within each analysis, i.e., taking into account genome size differences and ploidy level (Table 3). Like in individual analyses, only clusters exceeding the threshold of 0.01% were annotated at the supercluster level. Cluster abundances were analyzed in two ways (see Macas et al., 2011; Renny-Byfield et al., 2013; McCann et al., 2018): (1) by comparing their absolute sizes (in read numbers), and (2) by calculating a ratio of cluster abundances between samples, which removes cluster size effect. In the case of (1), we statistically assessed whether overall cluster abundances of each natural and synthetic hybrid are closer to either parent. For this analysis, we first recalculated abundances of natural triploids to correspond to the monoploid genome size (1Cx) in order to make them comparable to homoploid genome size (1C) of diploid parents. Then, we used the absolute difference in the number of reads between parent 1 and hybrid and compared it to the absolute difference in the number of reads between parent 2 and hybrid. We applied a pairwise non-parametric Wilcoxon test on all differences across all clusters, separately for each parental-hybrid combination. We assessed the degree of ‘intermediacy’ (Difference_Parent1–Hybrid_ – Difference_Parent2–Hybrid_) across all parent1-hybrid-parent2 combinations and determined which hybrid type (natural or synthetic) has a more ‘intermediate position’ using a Wilcoxon test. In the case of (2) we compared the actual cluster abundances of natural polyploid apomictic and synthetic diploid F1 hybrids with the theoretically expected abundances, which were produced by summing up the number of reads of the parental samples in each cluster and taking into account the actual genome dosage (i.e., 2 Int : 1 Pre for *H. pallidiflorum*, 1 Int : 2 Pre for *H. picroides* and 1 Int : 1 Pre for F1 hybrids). For this purpose, deviation scores for all clusters were calculated following Renny-Byfield et al. (2013), using the formula: (observed cluster size/expected cluster size) - 1. Cumulative deviation scores were calculated for each sample and compared among them. In order to check for a significant difference between natural and synthetic hybrids, we compared the deviation scores’ means and the magnitude of variation between groups of samples using ANOVA and Levene test. Finally, individual clusters showing the highest deviation from the expected values were identified and evaluated for patterns of deviation across samples that could be group-specific. The reliability of their abundance was double-checked by BLASTn searches (version BLAST+ 2.6.0; Altschul et al., 1990) of the initial, quality- and chloroplast/mitochondrial-filtered NGS datasets using their contigs as queries. The BLASTn parameters followed those in RE for assembling clusters.

**TABLE 3:** Combinations of samples subjected to comparative RepeatExplorer analysis.

Sample IDs and numbers of reads	Total no. of reads	Coverage^a^
**a) Comparison of the representatives of parental species**		
IntX^b^ (1,622,720) + PreX^c^ (1,544,840)	3,167,560	0.066
**b) Comparison of natural apomictic hybrids (3x) and representatives of parental species**		
PalA (2,438,920) + IntX^b^ (1,622,720) + PreX^c^ (1,544,840)	5,606,480	0.066
PalF (2,492,160) + IntX^b^ (1,622,720) + PreX^c^ (1,544,840)	5,659,720	0.066
PicF (2,455,200) + IntX^b^ (1,622,720) + PreX^c^ (1,544,840)	5,622,760	0.066
PicB (2,293,280) + IntX^b^ (1,622,720) + PreX^c^ (1,544,840)	5,460,840	0.066
**c) Comparison of synthetic hybrids (2x) and their actual parents**		
Hyb2 (1,920,000) + IntA (1,968,000) + PreC (1,872,000)	5,760,000	0.080
Hyb3 (1,920,000) + IntA (1,968,000) + PreC (1,872,000)	5,760,000	0.080
Hyb4 (1,914,668) + IntA (1,968,000) + PreC (1,872,000)	5,754,668	0.080

*^*a*^Coverage calculated using 1C-values.^*b*^IntX: sample created by pooling reads from both *H. intybaceum* individuals.^*c*^PreX: sample created by pooling reads from both *H. prenanthoides* individuals.*

### Development and Application of New Satellite Probes

Altogether, besides rDNAs, six satellite repeats were detected in *Hieracium* genomes using RE. Three of them were already described and used as FISH probes in Belyayev et al. (2018). We used contigs of the remaining three new satellites (Supplementary Figure 1) as templates for development of new FISH probes and tested their usability as cytogenetic markers.

The first novel satellite repeat, originally detected as cluster (CL) 229 in IntA, is a perfect inverted repeat of 46 bp, a sequence unsuitable for PCR amplification. Instead, half of it was synthetized as a 23 bp modified oligonucleotide (AAGACTTATACACATCCAAGAAG) labeled with Cyanine3 (Cy3) at the 5’-end (Eurofins Genomics, Ebersbach, Germany); FISH probe Cy3-CL229.

For the second satellite repeat, CL217 (a 126 bp monomer), contigs of 378 bp from nine *Hieracium* samples as well as all repeats contained within each contig were aligned in BioEdit (Hall, 1999). Primers int_126F (CTAAATGTTGC ATCATGTTCG) and int_126R (TGTATGATCCA CGGAATGC) were designed toward conserved motifs and employed for PCR amplification under the following conditions: 25-μl reaction mixtures contained 2.5 μl of 10 × PCR Blue buffer T059, 2.5 mM MgCl_2_, 0.2 mM of each primer, 200 μM of each dNTP, 20 ng DNA of IntA and 0.5 U *Taq* DNA polymerase (Top-Bio, Vestec, Czechia). The temperature profile included an initial denaturation step at 95°C for 5 min, followed by 35 cycles consisting of 95°C for 30 s, 50°C for 30 s, 72°C for 1 min and a final extension step at 72°C for 15 min. Amplification produced a ladder typical for tandem repeats. The longest bands were cut, purified, cloned, re-amplified and sequenced as described in Belyayev et al. (2018). Cloned sequences were aligned with the contigs, and one clone of 243 bp length (GenBank accession number MN784126) corresponding to almost two full repeats was used for FISH probe preparation (probe int126X2).

For the third satellite repeat, CL201 (a 172 bp monomer), aligned repeats of the longest contig (642 bp) of PreA were used for primer design; primers Hpre201f (ACTGGTCTCAAAT GCTTAGG) and Hpre201r (AAGCATTTGAGACCAGTAGG) were used for PCR amplification under the same conditions like above with the following alterations: 1.5 mM MgCl_2_ and DNA of PreA were used, annealing temperature was 52°C, and extension at 72°C was done for 2 min. Subsequent procedures were the same as described above, and one clone (GenBank accession number MN784127) consisting of almost three full repeats (533 bp) was chosen for FISH probe preparation (Hpre201X1).

Chromosome preparation and *in situ* hybridization procedures were done as described in Belyayev et al. (2018). Hpre201X1 was found to co-localize with 5S rDNA in previously tested accessions of *H. prenanthoides* (data not shown), therefore we sequenced the non-transcribed spacer (5S-NTS) of two samples of each *H. intybaceum* and *H. prenanthoides* following Kaplan et al. (2013) to see if they may correspond with the tandem repeat. 5S-NTS sequences (GenBank accession no. MN784128-MN784131) were different from the repeat probe, therefore Hpre201X1 is localized near the 5S locus of those accessions, but not a part of it.

All accessions included in this study were tested with FISH probes of the following tandem repeats: 45S rDNA, 5S rDNA, CL82 (369 bp), and newly developed CL229 (46 bp), CL217 (126 bp) and CL201 (172 bp). The satellites CL18 (23+21 bp) and CL160 (89 bp) were excluded from the FISH experiments, because in preliminary trials they were found to be uninformative (data not shown). The results were compared with the results of RE and BLAST searches of the entire NGS datasets.

## Results

### Bias Due to Library Preparation and Similarity Between Parental Accessions

**FIGURE 1:**
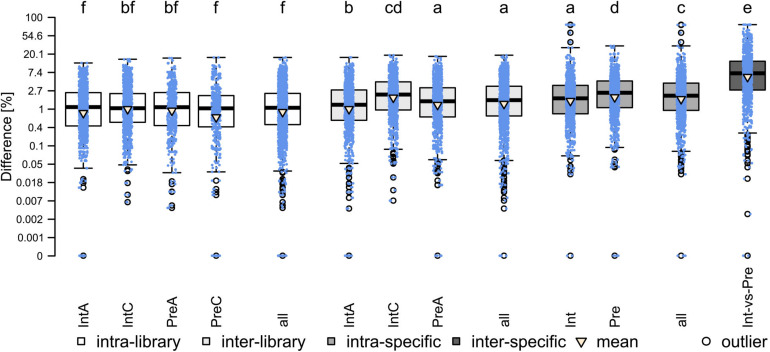
Results of testing the bias due to library preparation and similarity between parental accessions (paired Wilcoxon signed-rank test). Different letters indicate significant differences between groups. Mean values (triangle) from left to right: 1.89, 1.96, 1.97, 1.83, 1.92, 2.15, 3.12, 2.41, 2.56, 3.72, 3.47, 3.60, 11.78.

The results of the analysis are presented in Figure 1. The average subsampling bias (intra-library) was the lowest (mean: 1.92%), followed by the library preparation bias (inter-library; mean: 2.56%) the comparison of different accessions of the same species (intra-specific; mean: 3.60%) and the bias due to parental accessions (inter-specific, mean: 11.78%). All those differences were highly significant (*p* < 0.001). As for the inter-library comparisons, IntC (mean: 3.12%) showed significantly higher (*p* < 0.001) bias than the libraries of IntA (mean: 2.15%) and PreA (mean: 2.41%), which, in turn, had the same magnitude as the intra-specific comparison of Pre (mean 3.47%). Overall, despite being significantly different, the magnitude of library preparation bias falls within the magnitude of subsampling bias and the magnitude of intraspecific variation. Therefore, we assume that library preparation does not affect conclusions for our interspecific comparisons.

### Individual Clustering and Annotation of Genomes

**FIGURE 2:**
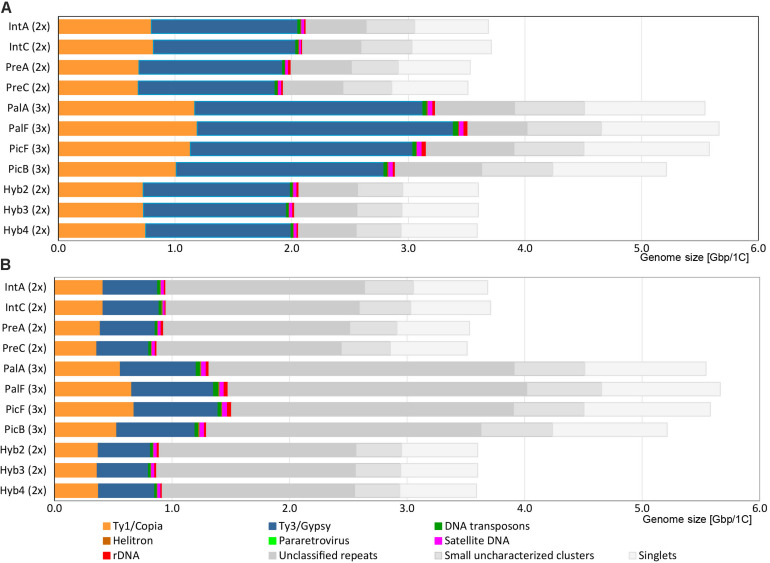
Genomic composition of *Hieracium* species. **(A)** Results of annotation at the supercluster level. **(B)** Results of annotation at the cluster level. Repetitive elements like pararetrovirus and Helitrons are present in amounts too low to be visible on the graphs.

In the individual clustering analyses, the pipeline used 3.7–4.8 million reads per sample, which corresponds to a genomic coverage of 0.17–0.20 x for diploids and 0.10–0.12 x for triploids (Table 2). On average, the majority of reads were grouped into ca. 140,000 clusters composed of 2 reads and more, corresponding to ca. 82% of the genomes (Table 2), while singlets represented the remaining 18% of the genomes. About 70% of all reads belonged to clusters containing at least 0.01% of the total input reads, representing medium and highly abundant repetitive elements (in total 209–260 clusters). The annotation at the level of superclusters assigned on average 70% of clusters (total of 55–62% of reads) to specific types of repetitive elements while the rest of clusters containing 9–15% of the input reads remained unclassified (Figure 2A and Table 4). The repeatomes of all genomes were dominated by LTR retrotransposons, constituting 53–60% of the whole genome (Figure 2A, Table 4, and Supplementary Tables 1, 2). The LTR Ty1/Gypsy elements occupied 33–39% of the genomes and were represented by four clades: Tekay and CRM (chromovirus lineages) and Athila and Ogre Tat (non-chromovirus lineages). The Tekay clade dominated, ranging from 32 to 36% of the genome. In all accessions, the majority of Tekay clusters were connected into one dominant supercluster (no. 1; consisting of 29–45 clusters, which on average contained 25% of all input reads (Supplementary Figure 2). Athila occurred at 1.35–1.80%, while CRM and Ogre Tat were present only in trace amounts. The LTR Ty1/Copia elements belonged to seven families and occupied 19–22% of the genomes. Among them, the most abundant was Maximus/SIRE, and with 14–17% represented the second most abundant repeatome component. The Angela family of Ty1/Copia was moderately abundant, and its content was constant across all samples (average 4.3%). Other Ty1/Copia families–Ale, Bianca, Ikeros, TAR and Tork constituted all together only ca. 1% of the *Hieracium* genomes. DNA transposons on average covered only 0.65% of the genomes with four types: EnSpm_CACTA, hAT, MuDR_Mutator and PiF_Harbinger. Other dispersed repetitive elements included Helitrons and pararetroviruses. The latter elements were not detected at the 0.01% threshold in *H. prenanthoides*.

**TABLE 4:** Results of annotation at the supercluster and cluster level.

Sequence type	Repeat family	Genomic proportion (%)^a^										
		IntA	IntC	PreA	PreC	PalA	PalF	PicF	PicB	Hyb2	Hyb3	Hyb4
Ty1/Copia retrotransposons	Ale	0.149	0.149	0.177	0.176	0.147	0.151	0.192	0.152	0.159	0.160	0.154
	Angela	4.199	4.202	4.474	4.409	4.202	4.334	4.408	4.397	4.351	4.319	4.363
		*(2.740)*	*(2.829)*	*(3.055)*	*(2.864)*	*(3.196)*	*(3.319)*	*(3.426)*	*(3.050)*	*(2.799)*	*(2.951)*	*(2.845)*
	Bianca	0.163	0.163	0.155	0.164	0.165	0.169	0.160	0.172	0.164	0.166	0.169
	Ikeros	0.032	0.030	0.015	0.000	0.015	0.017	0.000	0.018	0.017	0.016	0.017
	SIRE	16.399	16.776	14.043	14.151	15.940	15.734	14.873	14.022	14.874	14.929	15.535
		*(7.442)*	*(7.328)*	*(6.926)*	*(6.466)*	*(5.981)*	*(7.335)*	*(7.719)*	*(6.134)*	*(6.571)*	*(6.114)*	*(6.654)*
	TAR	0.337	0.277	0.257	0.254	0.258	0.267	0.292	0.276	0.265	0.266	0.255
	Tork	0.367	0.359	0.470	0.346	0.359	0.351	0.354	0.351	0.360	0.431	0.356
	**total Ty1/Copia**	**21.646**	**21.956**	**19.591**	**19.500**	**21.086**	**21.023**	**20.279**	**19.388**	**20.190**	**20.287**	**20.849**
		*(11.230)*	*(11.135)*	*(11.055)*	*(10.270)*	*(10.120)*	*(11.609)*	*(12.143)*	*(10.153)*	*(10.335)*	*(10.104)*	*(10.450)*
Ty3/Gypsy retrotransposons	chromovirus/Tekay	31.687	30.830	33.006	31.413	33.114	36.522	32.350	32.104	32.849	31.884	32.571
		*(10.227)*	*(10.843)*	*(11.456)*	*(10.583)*	*(9.570)*	*(10.049)*	*(10.997)*	*(10.980)*	*(10.156)*	*(9.983)*	*(11.269)*
	chromovirus/CRM	0.014	0.029	0.032	0.064	0.023	0.027	0.000	0.063	0.026	0.095	0.039
	non-chromovirus/OTA/Athila	1.773	1.472	1.355	1.502	1.668	1.699	1.380	1.528	1.667	1.567	1.622
		*(1.756)*	*(1.457)*	*(1.335)*	*(1.483)*	*(1.652)*	*(1.699)*	*(1.363)*	*(1.510)*	*(1.667)*	*(1.548)*	*(1.602)*
	non-chromovirus/OTA/Ogre_Tat/TatV	0.571	0.527	0.463	0.441	0.482	0.512	0.445	0.453	0.500	0.482	0.491
		*(0.571)*	*(0.527)*	*(0.375)*	*(0.441)*	*(0.398)*	*(0.512)*	*(0.445)*	*(0.239)*	*(0.500)*	*(0.482)*	*(0.409)*
	**total Ty3/Gypsy**	**34.045**	**32.858**	**34.856**	**33.420**	**35.287**	**38.760**	**34.175**	**34.148**	**35.042**	**34.028**	**34.723**
		*(12.568)*	*(12.856)*	*(13.198)*	*(12.571)*	*(11.643)*	*(12.287)*	*(12.805)*	*(12.792)*	*(12.349)*	*(12.108)*	*(13.319)*
pararetrovirus		0.069	0.055	0.000	0.000	0.050	0.031	0.017	0.042	0.021	0.029	0.031
DNA transposons	EnSpm_CACTA	0.175	0.178	0.145	0.177	0.163	0.265	0.284	0.168	0.151	0.189	0.160
	hAT	0.068	0.056	0.068	0.063	0.070	0.060	0.060	0.075	0.058	0.034	0.060
	MuDR_Mutator	0.152	0.159	0.149	0.184	0.159	0.195	0.090	0.073	0.160	0.092	0.089
		*(0.132)*	*(0.159)*	*(0.148)*	*(0.167)*	*(0.159)*	*(0.195)*	*(0.090)*	*(0.073)*	*(0.160)*	*(0.092)*	*(0.089)*
	PIF_Harbinger	0.262	0.284	0.286	0.284	0.241	0.253	0.123	0.283	0.279	0.256	0.249
	**total DNA transposons**	**0.657**	**0.677**	**0.648**	**0.708**	**0.633**	**0.773**	**0.557**	**0.599**	**0.648**	**0.571**	**0.558**
		*(0.636)*	*(0.677)*	*(0.647)*	*(0.691)*	*(0.633)*	*(0.773)*	*(0.557)*	*(0.599)*	*(0.648)*	*(0.571)*	*(0.558)*
Helitron		0.160	0.155	0.105	0.133	0.143	0.125	0.101	0.146	0.126	0.124	0.128
satellite DNA	satellite CL18 ^b^ (23+21 bp)	0.405	0.349	0.369	0.424	0.471	0.414	0.477	0.485	0.417	0.452	0.405
	satellite CL160 (89 bp)	0.129	0.125	0.118	0.089	0.113	0.119	0.088	0.114	0.108	0.121	0.124
	satellite CL82 (369 bp)	0.086	0.000	0.163	0.200	0.077	0.103	0.120	0.106	0.192	0.112	0.108
	satellite CL229 (46bp)	0.042	0.000	0.000	0.000	0.023	0.014	0.021	0.021	0.024	0.025	0.025
	satellite CL201 (172bp)	0.000	0.000	0.026	0.028	0.000	0.000	0.031	0.013	0.025	0.014	0.027
	satellite CL217 (126bp)	0.023	0.029	0.000	0.011	0.016	0.021	0.013	0.018	0.014	0.017	0.023
	**total satellite DNA**	**0.685**	**0.503**	**0.676**	**0.752**	**0.700**	**0.671**	**0.749**	**0.758**	**0.781**	**0.741**	**0.712**
45S rDNA		0.331	0.225	0.576	0.380	0.394	0.561	0.619	0.297	0.458	0.442	0.331
5S rDNA		0.027	0.000	0.052	0.024	0.029	0.028	0.021	0.024	0.031	0.030	0.023
unclassified repeats		13.918	13.445	14.613	14.562	12.211	8.952	13.447	14.202	13.918	14.836	13.755
		*(45.831)*	*(44.267)*	*(44.806)*	*(44.658)*	*(46.821)*	*(44.839)*	*(42.954)*	*(44.793)*	*(46.465)*	*(46.939)*	*(45.557)*
small uncharacterized clusters		11.336	11.817	11.436	11.938	10.856	11.264	10.779	11.707	10.797	10.733	10.717
singlets		17.128	18.311	17.450	18.582	18.612	17.811	19.256	18.690	17.988	18.179	18.172

*^*a*^Values in regular font represent results of annotation at the supercluster level; results at the cluster level are indicated in *italics* and in parentheses. Annotations at the cluster level are only reported when they differ from the ones at the supercluster level.^*b*^complex structure with nested repeats including two minisatellites (see Belyayev et al., 2018).*

The results of the annotation at the supercluster level were then compared with the annotation at the level of clusters, which left many clusters unannotated (Table 4, values in parentheses). The cluster level approach annotated less than half of the clusters (total of 25–27% of input reads), while the remaining unannotated ones containing ca. 43–47% of reads had few or completely lacked protein domain BLAST hits (Figure 2B and Supplementary Table 2). The main drivers for the difference in annotation between supercluster and cluster level were Tekay, Maximus/SIRE and Angela. These were highly abundant TEs that formed several large superclusters composed of both annotated and unannotated clusters (Table 4 and Supplementary Tables 1, 2). Ty3/Gypsy Athila, Ogre/Tat and MuDR_Mutator type of DNA transposons also formed several smaller superclusters. Other TEs did not form superclusters, and therefore, their genomic proportion remained the same in both approaches.

### Comparative Clustering Analysis of Parental Species, Natural, and Synthetic Hybrids

**FIGURE 3:**
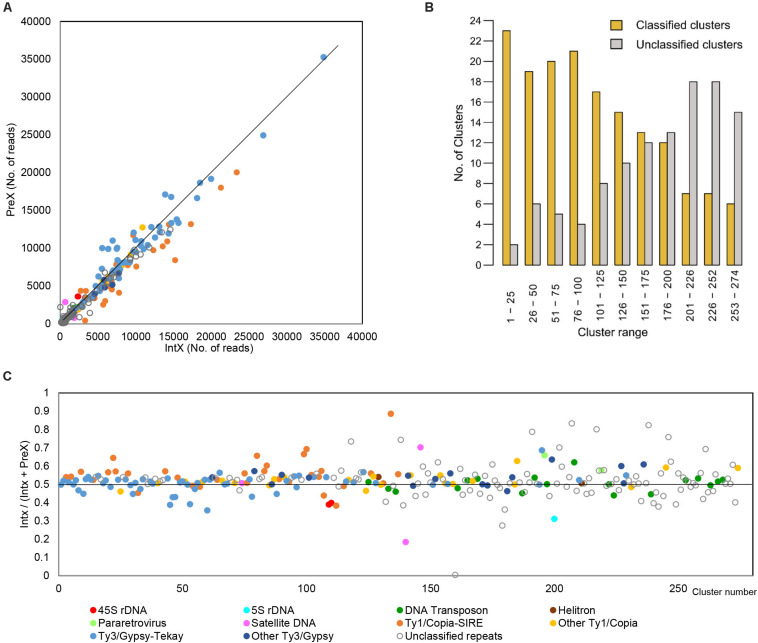
Results of comparative analysis of the two parental species. **(A)** Comparison of the number of reads from *H. intybaceum* (IntX) and *H. prenanthoides* (PreX) across all clusters. Dots represent clusters of repetitive elements. Clusters placed on the line represent repeats with the same genomic proportion in both species. **(B)** Proportion of annotated (classified) and unannotated (unclassified) clusters. Clusters are arranged by decreasing size. **(C)** Comparison of cluster abundances in *H. intybaceum* (IntX) and *H. prenanthoides* (PreX) irrespective of cluster size. Clusters on the black line (value of 0.5) have the same proportion in both species; clusters above are more abundant in *H. intybaceum*; clusters below are more abundant in *H. prenanthoides*.

The comparative analysis of the parental species *H. intybaceum* (IntX) and *H. prenanthoides* (PreX) revealed a high similarity in abundance across all major repeat types (Figure 3A). The largest clusters that contributed the highest to the genome size variation between species contained mostly chromovirus Tekay (slightly more abundant in *H. prenanthoides*) and Maximus/SIRE (more abundant in *H. intybaceum*). However, after removing the effect of cluster size (Figure 3C), the highest variability occurred among small clusters that contribute the least to the total genome size variation (Figure 3C). The most variable elements were unclassified repeats. Overall, the number of annotated clusters decreased with cluster size (Figure 3B).

**FIGURE 4:**
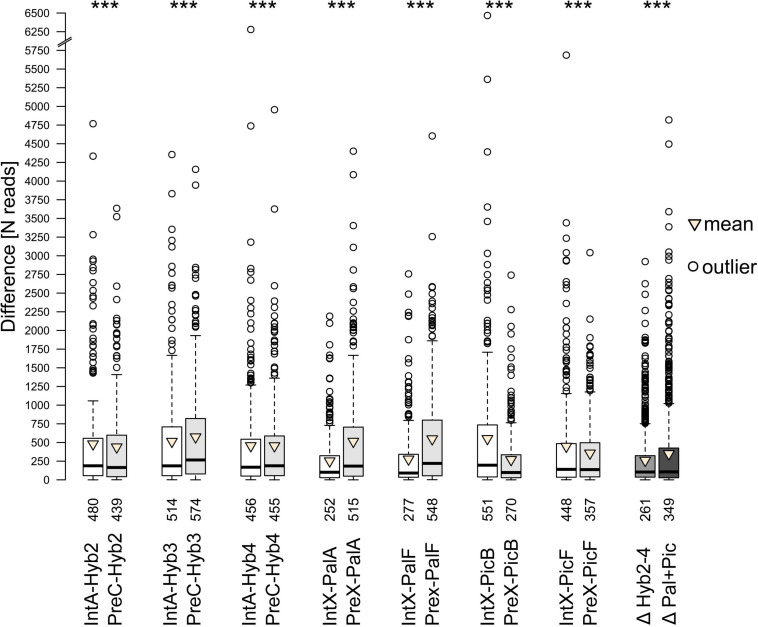
Difference in number of reads between parental and progeny accessions obtained from comparative analyses. The paired Wilcoxon signed-rank test was, separately for each parent-progeny combination, employed to access whether the progeny is closer to either parent. Results are shown above the boxes. Numbers below the boxes indicate mean differences in read numbers for the respective comparison. Δ – Degree of ‘intermediacy’ of progeny (ΔParent1-Progeny – ΔParent2-Progeny). Significance level *** – *p* < 0.001.

In the comparative analyses of parental species and their natural and synthetic hybrids, we compared firstly their cluster abundances in read numbers (Supplementary Figure 3). In all hybrids, the majority of clusters showed an intermediate position between the clusters of parental species. However, across all comparisons, each hybrid was significantly closer to one of the parents (Figure 4). Cluster abundances of PalA and PalF were significantly more similar to Int, while PicB and PicF were significantly more similar to Pre. This observation reflects well the parental genome dosages of 2:1 and 1:2, respectively. The position of the clusters of synthetic hybrids was more intermediate when compared to natural hybrids. No cluster was specific for any of the hybrids or parental species except for the occasional presence/absence of a certain satellite repeat.

**FIGURE 5:**
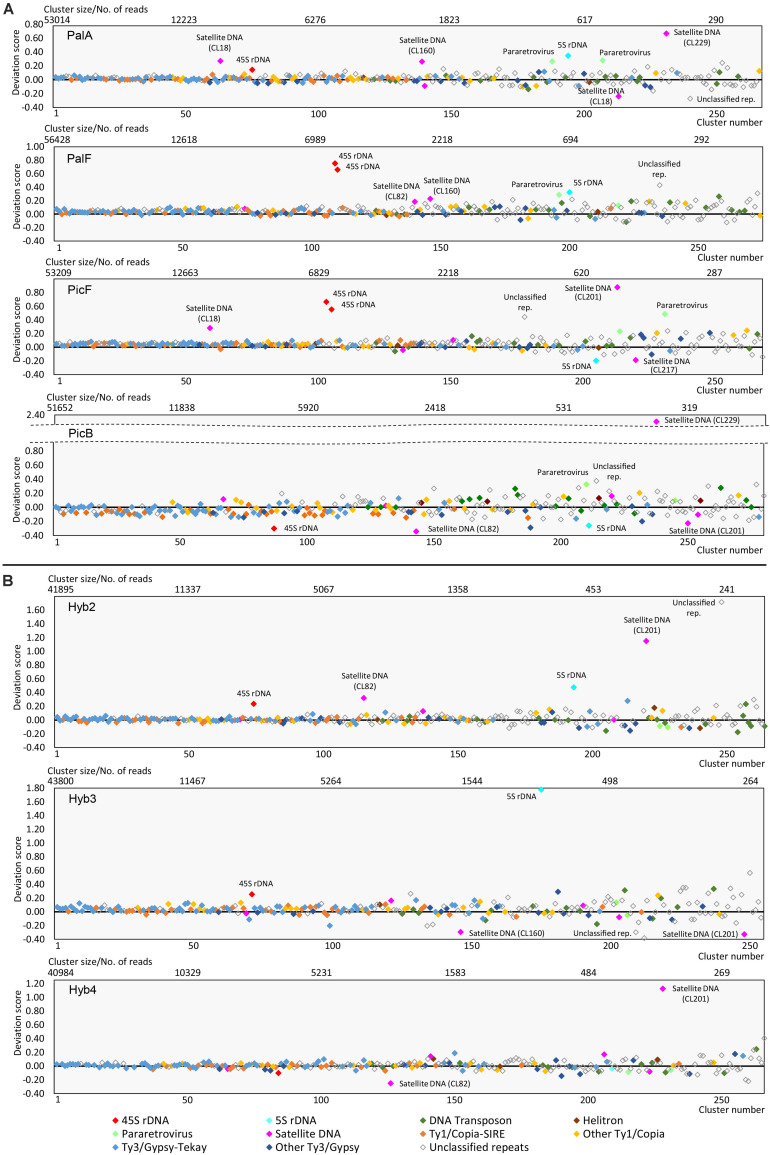
Deviation of clusters from their expected size in: **(A)** natural triploid apomicts *H. pallidiflorum* (PalA, PalF) and *H. picroides* (PicF, PicB) and **(B)** synthetic diploid hybrids (Hyb2, Hyb3, Hyb4). Clusters are arranged by decreasing size. The symbols on the black line (value zero) indicate clusters of expected cluster size. Clusters above or below this line are larger or smaller compared to the expected values calculated from the parental species.

Theoretically, in a newly formed hybrid, we would expect a direct inheritance of the repeat content from the parents, in proportions that correspond to the genome dosage of the hybrid. Significant deviation from this initial content in synthetic and natural hybrids would indicate a burst (or loss) of TEs. We compared the observed cluster abundances of F1 hybrids and natural allopolyploids with the expected values that were calculated based on the parental abundances. As a measure of departure from expectations, we used deviation scores, by which the cluster size effect is removed (Figure 5 and Supplementary Figure 4). A positive deviation score implies a larger cluster size than expected, and vice versa for a negative deviation score (Figure 5).

In general, the majority of clusters of all hybrids follow the theoretical expectation. The deviation tends to be the smallest in big clusters and increases as clusters get smaller. In PalF, PicF and Hyb3, the majority of clusters were slightly more abundant than expected (Supplementary Figure 4, ascending curve). In PicB, clusters were more dispersed, and the largest clusters (the first hundred clusters) were slightly smaller than expected (Supplementary Figure 4, descending curve). The clusters of PalA, Hyb2 and Hyb4 were the closest to the expected values. The statistical assessment of overall deviation scores’ means and the magnitude of variation of deviation scores in two groups of accessions (natural vs. synthetic) showed no statistically significant difference between natural apomictic and synthetic hybrids (ANOVA: *F* = 0.561, *p* > 0.05; variation of deviation scores - Levene test: *F* = 0.8221, *p* > 0.05).

Nevertheless, 45S rDNA, 5S rDNA and several satellite repeats deviated substantially from the general trend (Figure 5). Consistent deviation was found for 45S rDNA and 5S rDNA across all samples except Hyb4. However, they displayed sample specific trends with either under- or over-representation. The over-representation of 45S rDNA is the strongest in PalF and PicF, which contained the highest amount of 45S rDNA in all hybrids in the individual clustering analysis (Table 4). Consistent deviation was also found for satellite CL201 (172bp), which deviated from expectation in Pic and in synthetic hybrids (in Pal this satellite was not detected). Other satellite repeats did not show any strong pattern of deviation across groups of samples (natural hybrids vs. synthetic hybrids; Pal vs. Pic; Hyb vs. Pal or Pic). An occasional deviation of a few unclassified repeats was detected, which also did not show any consistent pattern.

In contrast, all apomicts displayed a higher deviation (an increase) of pararetrovirus clusters than synthetic hybrids. This represents the only apomict-specific finding among clusters of repetitive elements. In all comparative analyses, two pararetrovirus clusters were detected. In PalA and PicF accessions, both clusters were considerably larger than expected (deviation scores in PalA: 0.263 and 0.278; PicF: 0.230 and 0.486), whereas in PalF and PicB, only one of the two clusters showed a prominent deviation (PalF: 0.289; PicB: 0.328). Statistical analyses (Supplementary Figure 5) showed the differences between natural and synthetic hybrids to be highly significant (mean deviation scores: synthetic hybrids −0.04, natural hybrids 0.26; ANOVA: *F* = 27.2; *p* < 0.001).

### Abundance and Cytogenetic Analyses of rDNA and Satellite Repeats

45S rDNA content varied substantially between accessions (0.225–0.619%). The average content of 5S rDNA was only 0.029%, and it was not detected at all in IntC (but see below). According to the individual RE clustering, satellite DNA represented only a tiny fraction of *Hieracium* genomes (from 0.503% in IntC to 0.781% in Hyb2) (Table 4). In addition to the three described satellite repeats in *Hieracium* [CL18, CL82 and CL160 (Belyayev et al., 2018)], we detected three novel repeats, CL229, CL201 and CL217 (Supplementary Figure 1). CL18 and CL160 were detected by RE in all accessions, while CL82 was detected in all accessions except in IntC (Table 5). Satellite CL229 (46 bp) was only detected in IntA and in all natural and synthetic F1 hybrids, but not in IntC and both Pre accessions. CL201 (172 bp) was detected in both Pre accessions, but not in any of Int; it was also found in Pic and synthetic hybrids, but not in Pal accessions. CL217 (126 bp) was detected in all accessions except PreA.

**TABLE 5:** rDNA and satellite repeats as detected by RepeatExplorer, BLASTn and FISH.

	45 rDNA			5S rDNA			CL82 (369 bp)			CL229 (46bp)			CL217 (126 bp)			CL201 (172bp)^e^		
ID	RE (+/−)	BLAST (+/−)	No. of FISH loci	RE (+/−)	BLAST (+/−)	No. of FISH loci	RE (+/−)	BLAST (+/−)	No. of FISH loci	RE (+/−)	BLAST (+/−)	No. of FISH loci	RE (+/−)	BLAST (+/−)	No. of FISH loci	RE (+/−)	BLAST (+/−)	No. of FISH loci
IntA	+	+	4	+	+	2	+	+	1	+	+	2	+	+	2	–	–	0
IntC	+	+	4	–	+	2	–	(+)^b^	0	–	(+)^c^	0	+	+	2	–	–	0
PreA	+	+	6	+	+	2	+	+	7	–	(+)^c^	0	–	+	0	+	+	2
PreC	+	+	6	+	+	2	+	+	7	–	(+)^c^	0	+	+	0	+	+	2
PalA	+	+	7	+	+	3	+	+	2	+	+	1	+	+	2	–	(+)^c^	1
PalF	+	+	7	+	+	3	+	+	6	+	+	1	+	+	3+6^d^	–	(+)^c^	0
PicF	+	+	7^a^	+	+	3	+	+	9	+	+	1	+	+	4+8^d^	+	+	2
PicB	+	+	7^a^	+	+	3	+	+	6	+	+	1	+	+	2	+	+	2
Hyb2	+	+	5	+	+	2	+	+	4	+	+	1	+	+	1	+	+	2
Hyb3	+	+	5	+	+	2	+	+	4	+	+	1	+	+	1	+	+	1
Hyb4	+	+	5	+	+	2	+	+	6	+	+	1	+	+	1	+	+	2

**FIGURE 6:**
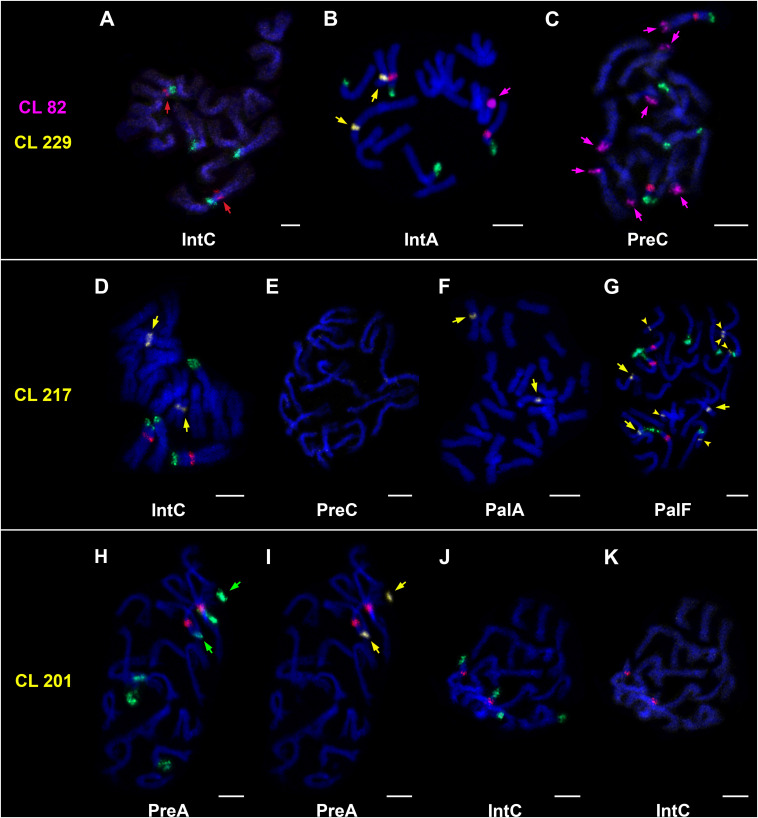
Chromosomal localization of clusters/satellites by FISH. 5S rDNA (red signal), 45S rDNA (green signal). **(A–C)** Presence/absence of 5S rDNA (**A**, red arrows), CL82 (**B,C**, magenta arrows) and CL229 (**B**, yellow arrows). **(D–G)** Presence/absence of CL217 (**D,F,G** – major loci marked by yellow arrows; **G**–additional loci marked by yellow arrowheads). **(H–K)** Presence/absence of CL201. The same metaphase plates are used in H/I to show co-localization of 45S rDNA (**H**, green arrows) and CL201 (**I**, yellow arrows), and J/K (absence of CL201). Chromosomes were counterstained with DAPI (blue). Bars = 5 μm.

*ID = Sample ID; RE/BLAST (+/−) = RepeatExplorer/BLAST (cluster detected/cluster not detected).^*a*^The numbers of loci for 45S rDNA in PicF and PicB were reported in Chrtek et al. (2020).^*b*^CL82 was detected in a very low amount in IntC (see Supplementary Table 3) and a closer inspection showed that the corresponding reads displayed a rather low similarity to the sequence of satellite CL82 (see section “Discussion”).^*c*^satellite repeat detected only in trace amounts (see Supplementary Table 3).^*d*^besides major loci found on all metaphase plates (PalF 3, PicF 4), additional loci (PalF 6, PicF 8) of CL217 occurred in these samples (see section “Results” and Figure 6).^*e*^CL201 was present as two hemizygous loci on non-homologous chromosomes in Pre.*

The results of cytogenetic experiments are shown in Figure 6 and Table 5. In short, FISH signals corresponding to 45S and 5S rDNA were detected in all accessions. CL82 was detected in all accessions except in IntC. The tandem structure of the three new satellites was confirmed cytogenetically; the probe CL229 produced signals in IntA and all natural and synthetic hybrids, while no signal was detected in IntC and both Pre accessions. CL201 was detected in Pre and all natural and synthetic hybrids except in PalF, and was not found in Int accessions. Lastly, CL217 was observed in both Int accessions and in all natural and synthetic hybrids, but not in any Pre accessions.

### Comparison of RE, BLAST, and Cytogenetic Results

rDNA and satellite repeats showed the highest deviation from the expected values in natural and synthetic hybrids in RE. In order to verify the genuine absence of satellites and rDNA, that were not identified with RE, we searched for them with BLASTn on the whole filtered NGS datasets (Supplementary Table 3) and compared the outcome with the results of the FISH experiments (see Table 5).

FISH for 45S rDNA showed four chromosomal loci in *H. intybaceum* (two per haploid genome) and six loci in *H. prenanthoides* (Figures 6A–C). However, the genomic proportions of 45S rDNA obtained by RE were different among individuals within each parental species, despite having the same number of loci (IntA: 0.331%, IntC: 0.225%; PreA: 0.576%; PreC: 0.380%). While all tested accessions of *H. pallidiflorum* and *H. picroides* bear the same number of 45S rDNA loci (7), the accessions differed in their genomic proportion detected by RE. Similarly, the three synthetic hybrids possessed the same number of loci (5), but they also differed in their proportions obtained by RE. The results of BLASTn searches for 45S rDNA were in a good agreement with the RE results.

As of 5S rDNA, all tested accessions had one FISH locus per haploid genome (i.e., all diploids had 2, whereas triploids had 3 loci) (Figure 6 and Table 5). Contrary to the FISH results, the proportion of 5S rDNA in PreA was twofold higher than in PreC (0.052% vs. 0.024%), and similarly, IntA had 0.027% (and a slightly higher result in BLASTn search of the entire NGS dataset) while IntC had only 0.13% of 5S rDNA, which might explain why it was not detected by RE (Figure 6A). In contrast to the parental species, both groups of natural and synthetic hybrids had relatively similar genomic proportions of 5S rDNA as detected by RE. The RE and BLASTn results showed slightly less congruence than other tested repeats.

Only one CL82 locus was detected by FISH in IntA, whereas IntC does not possess this tandem repeat according to FISH (Figures 6A,B) and RE; but the BLASTn search found 0.007% of reads corresponding to this repeat in IntC (below the RE threshold). The accessions of *H. prenanthoides* had 7 loci each (Figure 6C), however, their total copy numbers detected by RE were only about twice as high as in IntA. In natural apomicts, the number of CL82 loci was variable (Table 5 and Supplementary Figure 6), and the genomic proportions obtained by RE approximately followed the pattern detected by FISH (the smallest amount in PalA, the highest in PicF, approximately the same amount in PalF and PicB). The picture was not entirely consistent in synthetic hybrids; the genomic abundances detected by RE did not follow the number of FISH loci. RE and BLASTn results were generally in agreement.

The novel satellite repeat CL229 (46 bp monomer) was only found in one accession of *H. intybaceum* (IntA) and in all hybrids. In IntA, it was present in 2 loci on a pair of homologous chromosomes (Figure 6B). While the satellite was not detected in IntC nor in both *H. prenanthoides* by RE and FISH, BLASTn searches of their NGS datasets detected trace amounts of this repeat in these three accessions. All natural and synthetic hybrids had one CL229 locus each, with similar genomic proportions detected by both RE and BLASTn.

The novel satellite repeat CL217 (126 bp monomer) was detected by FISH in both accessions of *H. intybaceum* (2 loci on homologous chromosomes, Figure 6D), but not in *H. prenanthoides* (Figure 6E). However, RE and BLASTn of *H. prenanthoides* detected it in very small amounts: the NGS dataset of PreA contained 0.008% of the reads (BLASTn hits to this satellite repeat, no cluster detected by RE), whereas PreC contained 0.01% and a cluster detected by RE. Each of the synthetic hybrids contained one FISH signal. *Hieracium pallidiflorum* accessions had 2 or 3 major loci (Figures 6F,G), whereas *H. picroides* had 2 or 4 major loci. In one accession of each (PalF and PicF), a proliferation of this satellite was detected, resulting in 6 (Figure 6G) and 8 additional loci (Table 5), respectively. RE and BLASTn results were congruent.

Regarding the third novel satellite CL201 (172 bp monomer), two FISH loci were detected in each *H. prenanthoides* accession, but they did not constitute a homologous pair. Instead, they were hemizygous as indicated by their presence on chromosomes bearing or not bearing also 5S rDNA loci (Figures 6H,I). Furthermore, CL201 co-localized with 45S rDNA. Both accessions had similar genomic proportions according to RE and BLASTn. CL201 was not detected in *H. intybaceum* accessions by any of the methods (Figures 6J,K and Table 5). RE analyses did not detect CL201 in *H. pallidiflorum* accessions, nevertheless, BLASTn searches revealed trace amounts. Also, despite a low proportion of 0.005% in PalA, it produced a FISH signal; in PalF, the FISH locus was not detected. *Hieracium picroides* accessions possessed two loci each. The synthetic hybrids had one or two loci with correspondingly proportional genomic abundances.

## Discussion

### Characterization of *Hieracium* Repeatomes

This study represents the first characterization of repeatomes in *Hieracium* s.str. Transposable elements represent the dominant component of the investigated genomes, which is a general pattern in plants (Kejnovsky et al., 2012; Dubin et al., 2018). With 70% of TEs representing a genomic proportion of at least 0.01%, the *Hieracium* genomes can be considered as highly repetitive. The result is in accordance with a comparative study of the family Asteraceae (Staton and Burke, 2015) where the average proportion of TEs for the family was estimated to 69.9 ± 5.3% and the average proportions of LTRs and DNA transposons to 53 ± 19.1% and 0.60 ± 0.7%, respectively. *Hieracium* with an average of 55% for LTR retrotransposons and 0.65% for DNA transposons matches those results.

Staton and Burke (2015) revealed a linear increase in the abundance of Ty3/Gypsy elements from the most ancestral Asteraceae to the most derived subfamily, Asteroideae. Among the latter, species of the genus *Helianthus* and *Phoebanthus tenuifolius* exhibit the highest content of Ty3/Gypsy (62.4 ± 2.7% and 67.5 ± 5.6%, respectively). In contrast to Ty3/Gypsy, Ty1/Copia showed the opposite pattern: basal species in the family have proportionally more Ty1/Copia compared to species of Asteroideae. *Hieracium* species of the subfamily Cichorioideae fit this picture; Ty3/Gypsy (33%–39%) dominate over Ty1/Copia (19–22%), in a ratio of ca. 1.5–1.8 : 1. The closest representative of the subfamily Cichorioideae, the diploid *Taraxacum kok-saghyz*, exhibits a similar pattern for these two groups. However, other repeatome studies performed on *Helianthus* species (subfamily Asteroideae) reported a much higher variability in the ratio of Ty3/Gypsy and Ty1/Copia (1.45–5.91 : 1, Mascagni et al., 2017). Also, it seems that the dominance of Ty3/Gypsy over Ty1/Copia cannot be generalized to the whole subfamily Asteroideae; recent studies of *Melampodium* (McCann et al., 2018), *Anacyclus* and *Heliocauta* (Vitales et al., 2019) showed that some of their species are dominated by Ty1/Copia over Ty3/Gypsy. Those findings confirm the widely recognized trend of remarkable variation in repeat composition among related taxa, and with future studies, we expect to see even higher diversity in the repeat composition of Asteraceae.

In *Hieracium*, only four families of LTRs (Ty3/Gypsy chromovirus/Tekay and non-chromovirus/Athila, Ty1/Copia Angela and SIRE) were represented in amounts higher than 1%. Among those, Ty3/Gypsy chromovirus/Tekay and Ty1/Copia SIRE were by far the most abundant, present in a ratio of about 2:1. Again, these two lineages seem to prevail in the Asteraceae, although the ratios between them may differ (e.g., Mascagni et al., 2017; Mlinarec et al., 2019).

Among all LTRs detected, only these four most abundant families plus Ty3/Gypsy non-chromovirus/Ogre Tat (genomic proportion ca 0.5%) formed superclusters. The annotation at the supercluster level is clearly important, because it captured the proliferation and complexity of highly abundant TE families that together constitute about 54% of *Hieracium* genomes. The comparison of annotations at the supercluster and the cluster level showed that roughly one third of all clusters that lacked a BLAST match in the TE protein domain database were additionally annotated at the supercluster level due to pair-end reads shared with annotated clusters. Such a result is expected for large and repetitive genomes like *Hieracium*. Superclusters represent highly proliferated LTR families that comprise both active and inactive copies at various stages of degradation due to accumulated mutations: such a high sequence divergence causes a lack of BLAST similarity between copies of the same element and thus creates separate clusters (Novák et al., 2010). Since the NGS reads from different regions within the genome (containing both active and inactive copies) cluster together, it remains unclear to which extent such clusters and superclusters represent active elements. The splitting into clusters and superclusters is also a consequence of the exceptionally large size of all five LTRs (Neumann et al., 2019) and low coverage sequencing; low read depth and gaps in the coverage of particular sequences cause a lack of overlapping NGS reads that may otherwise fuse two or more clusters into one (Novák et al., 2010). Therefore, it is possible that an increase of the sequencing depth would facilitate the connection of clusters and foster additional annotations. Also, it should be emphasized that some of the clusters contained in superclusters likely comprise sequences that do not necessarily represent TEs (e.g., TE’s flanking regions). Their proportion is difficult to estimate. Taking this and other factors mentioned above into account, it means that an exact level of TE annotation cannot be achieved with low coverage sequencing. Therefore, genomic proportions of TE categories obtained by individual clustering that have similar values (Figure 2 and Table 4) in closely related/highly similar accessions cannot be strictly compared. Nevertheless, the intention of the method is to give a general estimation of repeatome components, and the supercluster approach ensures a good overview of the extent of proliferation of highly abundant TEs.

### TE Dynamics Following Hybridization and Polyploidization

Comparative clustering analysis evaluates efficiently the repeatome variability in parental taxa and their derived allopolyploids (Renny-Byfield et al., 2013; Dodsworth et al., 2017; McCann et al., 2018). In *Hieracium*, repeat abundances for synthetic and natural hybrids were remarkably similar to the expected values calculated from parental genomes. Overall, clusters of synthetic hybrids expectedly showed a high degree of adherence to the expected values. Rather unexpectedly, natural hybrids followed the same pattern, only with slight over- or under-representation of cluster abundances across all repeat categories that corresponded to individual differences in their genome sizes. Among TE clusters, there were no large bursts or eliminations of any particular TE that could be interpreted as a response to hybridization and/or polyploidization events. However, apomicts displayed evident overabundance of pararetrovirus clusters not observed in synthetic hybrids, and this is the only detectable difference between natural apomictic and synthetic F1 hybrids (Figure 5). The differences between these two groups were statistically highly significant (Supplementary Figure 5). This finding may point to the accumulation of pararetrovirus sequences following polyploidization. Pararetroviruses are a type of retroelements that have double-stranded DNA and use reverse transcription for their replication. They seem to be ubiquitous in plants, but it is not clear if they represent a neutral component of plant genomes (e.g., due to a potentially lost function) or if they represent pathogens or even contribute to virus resistance of the host (Staginnus et al., 2007). The dynamic behavior of pararetroviruses was also observed in a study of allotetraploid *Nicotiana tabacum*; one of the pararetrovirus families was more abundant in one of its progenitors, *N. tomentosiformis*, than in *N. tabacum*. Such a pattern is a result of either preferential elimination from the polyploid genome or specific accumulation in the diploid progenitor genome following polyploidization (Matzke et al., 2004). However, the significant overabundance of pararetrovirus clusters we detected in apomicts does not necessarily imply a causal relationship. Also, the actual parents of the natural hybrids are unknown and may have shown higher abundances of pararetrovirus sequences than the diploids used in this study (although to assume that just these two repetitive elements out of hundreds were affected in the observed way may not be very likely). Besides pararetroviruses, an occasional deviation of a few different unclassified repeats was observed, without any obvious pattern among accessions. However, both pararetrovirus and unclassified clusters are located among small clusters with increasing deviation from the expected values that creates the appearance of a ‘widening tail’ in the graph, which is a consequence of low-coverage sequencing. While it is possible that the higher variability of small clusters is a sign of ongoing evolutionary processes, the pronounced bias of low-coverage sequencing undermines definitive interpretations of these results.

Generally, our results corroborate the findings in two studies of Asteraceae genera using the graph-based clustering method, which also did not reveal differential amplification of TEs: allopolyploid species of *Melampodium* that most likely originated in the Pleistocene (McCann et al., 2018), and *Anacyclus*, which comprises diploid species of hybridogenous origin (Vitales et al., 2019). These findings are consistent with recent observations according to which amplification of TEs after hybridization and polyploidization seem to be rare (Parisod and Senerchia, 2012). It is possible that the hybridization event in our synthetic hybrids triggered TE activity, which resulted in transcriptional, epigenetic or structural changes that did not result in a net increase of TE copy number, and these aspects would be worth further investigation. In the case of *H. pallidiflorum* and *H. picroides*, it is not possible to predict possible long-term results of TE activity triggered by historical polyploidization events. Most probably, the observed repetitive profiles also depend on the age of the allopolyploids. It has been suggested that neopolyploidization events may be accompanied by transiently increased activity of TEs (release of TE silencing and possibly transposition) in the first generations of polyploids, after which TE silencing is restored (Vicient and Casacuberta, 2017), however, the response may vary between genomes and TE families. Also, polyploidization events that formed *H. pallidiflorum* and *H. picroides* might be immediately followed by a switch to apomixis so that no further generations could facilitate such restoration by meiosis. *Hieracium pallidiflorum* and *H. picroides* are assumed to be at least hundreds and up to several thousands of years old, and there is no evidence of a recent formation of polyploids by hybridization between diploid sexual taxa (Mráz and Zdvořák, 2019). So far, attempts of experimental crossings failed to produce triploid hybrids from the parental combination.

Concerning the mode of reproduction, there was no statistically significant difference between the groups of natural apomictic and synthetic *Hieracium* hybrids in the overall deviation pattern of their clusters from the expected values. This result is in agreement with a study of Ågren et al. (2015) on genus *Oenothera*, which comprises sexual species as well as species utilizing functionally asexual reproduction (permanent translocation heterozygosity); there was no evidence that the mode of reproduction was responsible for an almost twofold variation in genome size between the species. However, it should be emphasized that apomixis is considered a young evolutionary trait (Van Dijk and Vijverberg, 2005) and therefore it is possible that the consequences of asexuality in *Hieracium* are not yet detectable. This is in line with the study of Docking et al. (2006) where the authors did not find evidence of relaxed selection in three investigated types of TEs in four asexual taxa, among which were three presumably young Asteraceae: *Antennaria parlinii*, *Taraxacum officinale* and closely related *Hieracium aurantiacum* (recently reclassified as *Pilosella aurantiaca*; Bräutigam and Greuter, 2007).

The interesting diversity of genome sizes in natural apomicts (slight net increase in both *H. pallidiflorum* accessions; slight net increase or decrease in *H. picroides*), might be indeed a consequence of their different age and the independent evolution of TEs in each apomictic lineage after the polyploidization events, since all tested accessions were demonstrated to be of independent origin. However, other potential factors involved in the origin of these apomictic species that could produce the observed genome size differences cannot be ruled out. They include involvement of unknown (even extinct) diploid parental genotypes with higher genome size diversity, polyploid parental genotypes introgressed by other *Hieracium* species, and involvement of polyploid hybrids since they produce partially fertile pollen (Chrtek et al., 2020). In order to obtain more conclusive answers about the trends in genome size, measurements will have to be performed on a larger number of accessions of each apomictic and parental species. Overall, our results indicate a relatively low turnover of repetitive DNA during the formation of apomictic lineages and suggest that bursts of TEs do not play an important role in the evolution of our system.

### Dynamics of rDNA and Satellite Repeats in *Hieracium*

In contrast to TEs, rDNAs and satellite repeats showed substantial deviations from the expected values in both natural and synthetic hybrids, although because of their low abundance they do not significantly contribute to genome size differences. These deviations did not show any correlation with the accession origin, and are therefore explained by the hybridization event as such, independent of the ploidy and reproductive mode of the plants. Higher deviation of rDNA and satellite repeats over TEs was also reported in McCann et al. (2018) for allopolyploid species of *Melampodium*.

The RE findings for rDNA and satellite repeats were at first cross-checked with BLASTn searches of the entire NGS datasets. For the majority of satellite repeats and rDNAs, the genomic proportions detected by RE and BLASTn were congruent, but in a few occasions, different results were obtained. For example, 5S rDNA in IntC was not detected by RE, but BLASTn detected a genomic proportion of 0.013%. In contrast, the novel satellite repeat CL217 with its similar genomic proportion in PreC (0.01%) was detected by both RE and BLASTn. It should be noted that the low proportion of 5S rDNA in IntC is most likely not only a consequence of low-coverage sequencing bias as a similarly low proportion of this repeat was found in two independently sequenced libraries.

The overall picture became more complex when FISH results were compared with the results of RE and BLASTn: genomic abundance among accessions was not proportional to the number of FISH loci for the majority of repeats. This was especially pronounced in the case of 45S rDNA. That the number of loci does not necessarily correspond to the amount of tandemly repeated sequences contained in a locus was already shown in Rosato et al. (2016).

FISH experiments for the very low abundant satellite repeats (detected by BLASTn only) in several accessions also produced different results. The FISH loci were not detected for CL229 in IntC, PreA and PreC, and BLASTn found only trace amounts of this repeat. Probably its abundance may not have been high enough to be detectable by FISH. CL201 was present in a proportion of only 0.005% in PalA, but was sufficient to generate a FISH signal. In contrast, the satellite CL217 did not produce FISH signals despite its slightly higher genomic proportions in the genomes of PreA and PreC (0.008 and 0.01%, respectively). At this moment, it is not possible to answer why CL217 in PreA and PreC was not observed by FISH: most probably its abundance was below the discrimination level of the FISH protocol used (Valárik et al., 2004). CL82 in IntC had a similar genomic proportion detected by BLASTn (0.007%), but no FISH signal was detected. In contrast to other repeats, a closer inspection of this BLASTn finding showed that the corresponding reads displayed a rather low similarity to the sequence of satellite CL82 (maximum of 90% similarity over 60% of the read length only), which, in addition to its low abundance, possibly explains the lack of FISH signals.

The number of loci of the satellite repeats in hybrids (synthetic as well as natural) were in the range of expectation. In contrast to the genomic proportion of the satellites, the number of loci was not altered by hybridization and polyploidization events. The only exception was CL217: in one accession of each *H. pallidiflorum* (PalF) and *H. picroides* (PicF), a proliferation of this satellite was detected as additional minor loci. Moreover, additional minor loci were observed in the selfed progeny of one *H. intybaceum* accession (IntA; data not shown), which could be an indication that already at the intraspecific level, without the action of hybridization and polyploidization, satellites have a dynamic behavior. On the other hand, it could be explained as a consequence of differential chromatin condensation, which prevents the exposure of minor loci during FISH experiments (Krishnan et al., 2001).

Two satellite repeats displayed a hemizygous nature. Belyayev et al. (2018) have already shown CL82 as occurring in a hemizygous state in both parental species. Similarly, two non-homologous FISH loci of the novel marker CL201 were detected in *H. prenanthoides*; one locus was found on a chromosome bearing one 45S rDNA locus and the second on a chromosome bearing both 45S and 5S rDNA loci. Comparison of RE clusters of CL201 and rDNAs revealed that these repeats do not overlap. Furthermore, CL201 displayed intraspecific variation in its localization; in the case of two *H. prenanthoides* accessions, both CL201 loci co-localized with 45S only, but in repeated experiments on other accessions (data not shown), the repeat co-localized with 5S rDNA loci. The hemizygous pattern of CL201 in *H. prenanthoides* corresponds to the pattern subsequently observed in hybrids: their occurrence is in keeping with the genome dosage of *H. prenanthoides* in all natural and synthetic hybrids. This also explains why *H. pallidiflorum* had no (PalF) or one (PalA) locus. As this satellite presents two hemizygous loci, it can easily fail to get inherited, if the chromosome lacking it is passed on to the progeny. Hemizygous loci were reported in some apomictic species of *Pilosella* (Okada et al., 2011) and *Cenchrus* (Akiyama et al., 2005). In our case, the hemizygosity observed was not restricted to apomicts; it was already detected at the level of diploid sexual species and therefore, was not necessarily involved in the emergence of apomixis or facilitating a switch to this reproductive mode. Hemizygous loci can be caused by a hybridogenous origin of diploids (Myburg et al., 2003). Both parental species are in fact ancient hybrids; *H. intybaceum* most likely originated by wide hybridization between *Hieracium* and an unknown genus whereas *H. prenanthoides* shows signatures of a merger between Western and Eastern European lineages of genus *Hieracium* (Fehrer et al., 2007, 2009). The hybrid origin of many diploids in this genus may trigger polyploidization (linked with the apomictic mode of reproduction) as a means to stabilize the diploid hybrid genomes.

## Conclusion

Our results contribute to the understanding of plant genome evolution following hybridization and polyploidization and add to the knowledge about the genomic landscapes of polyploids known to undergo an asexual mode of reproduction. No evidence for a massive genome reorganization in TE abundance was found by studying repeatomes of parents, their natural apomictic and synthetic diploid hybrids, but a signal for a ‘genomic shock’ may still be present at the transcription or epigenetic level. The only elements showing deviations from expected abundances were two small pararetrovirus clusters that were significantly overrepresented in all apomicts. Whether there exists any causal relation to the mode of reproduction remains unclear, but this finding is worth further investigation. In contrast to TEs, satellite and rDNAs showed substantial deviations in all hybrids, independent of their ploidy, and were therefore a consequence of hybridization as such. Our study also highlights the need to study low-abundant repeats with a combination of approaches. We hypothesize that some deviations detected by different bioinformatic approaches may be a consequence of the rapidly evolving nature of these elements. Additional discrepancies between bioinformatic and cytogenetic evidence may in some cases be caused by bias in low coverage NGS or by a lack of sensitivity of *in situ* hybridization experiments. Therefore, results concerning low-abundant repeats should be interpreted with caution.

## Data Availability Statement

The datasets presented in this study can be found in online repositories. The names of the repository/repositories and accession number(s) can be found below: https://www.ebi.ac.uk/ena, PRJEB35856; https://www.ncbi.nlm.nih.gov/genbank/, MN784126–MN784131.

## Author Contributions

JF and DZ conceived of the study. DZ, JF, and YB designed bioinformatics experiments. DZ and MH analyzed the data. JF designed probes. LP and RS did cytogenetic analyses. JJ did molecular labwork. DZ wrote the manuscript. JF and YB suggested on structure and content. All authors contributed to the drafts and gave final approval for publication.

## Conflict of Interest

The authors declare that the research was conducted in the absence of any commercial or financial relationships that could be construed as a potential conflict of interest.

## Footnotes

1 https://repeatexplorer-elixir.cerit-sc.cz/galaxy/

2 http://sourceforge.net/projects/bbmap/

3 https://blast.ncbi.nlm.nih.gov/Blast.cgi

4 http://bioinfo.lifl.fr/yass/yass.php

## Supplementary Material

Supplementary Figure 1 Graphical representation of the newly detected satellite repeats. **(A)** RepeatExplorer graph layouts of the respective clusters showing a circular shape, typical for tandem repeats. **(B)** Self-similarity dot-plots of the cluster contigs created by the YASS genomic similarity search tool. The tandemly repeated motifs are displayed as green, parallel, diagonal lines, while the distance between lines equals lengths of the motifs. The red lines perpendicular to the main diagonal lines indicate inverted repeats.

Supplementary Figure 2 An example of summary histogram of RE clustering analysis in Hieracium (accession IntA). Superclusters are represented as columns in the histogram; the width of the columns corresponds to the size of superclusters. The horizontal lines within columns represent clusters in descending order of their sizes. The largest supercluster (no. 1) contains 23,14% of input reads and the first five superclusters contain 49% of all input reads.

Supplementary Figure 3 Comparative analyses of parental species and their natural and synthetic hybrids. **(A–D)** Comparative analyses of natural triploid hybrids and representatives of parental species (IntX - created by pooling reads from both individuals of *H. intybaceum*; PreX - created by pooling reads from both individuals of *H. prenanthoides.* **(E–G)** Comparative analyses of synthetic diploid hybrids and their actual parents. In **(A–D)**, the numbers of reads of triploids were recalculated using the monoploid genome size (1Cx), in order to make them comparable to homoploid genome size (1C) of diploid parents.

Supplementary Figure 4 Cumulative deviation scores of natural and synthetic *Hieracium* hybrids.

Supplementary Figure 5 Deviation scores of pararetrovirus clusters. Gray triangles show deviation scores of both pararetrovirus clusters detected in the seven comparative Repeat Explorer analyses. For combinations of samples subjected to comparative analyses, see text and Table 3. Deviation scores were grouped by hybrid origin (boxplots) and compared via ANOVA. The deviation score in pararetrovirus clusters was significantly higher in natural hybrids (mean deviation scores: synthetic hybrids −0.04, natural hybrids 0.26; ANOVA: *F* = 27.2; *p* < 0.001). ****p* < 0.01; syn – synthetic hybrids; nat – natural hybrids.

Supplementary Figure 6 Chromosomal localization of CL82 by FISH in synthetic and natural hybrids. **(A–C)** Synthetic F1 diploid hybrids. **(D–G)** Natural triploid hybrids. FISH signals: CL82 (purple signal, magenta arrows), 45S rDNA (green signal), 5S rDNA (red signal). Chromosomes were counterstained with DAPI (blue). Bars = 5 μm.

Supplementary Table 1 Individual clustering analyses – detailed annotation of individual clusters of all samples.

Supplementary Table 2 Summarized annotation at the supercluster (SCL) and cluster (CL) level, including numbers of clusters and superclusters.

Supplementary Table 3 Comparison of the most important RE individual clustering results with BLASTn searches of NGS datasets, in all accessions.
