# Supplementary material for: Characterization and Dynamics of Repeatomes in Closely Related Species of Hieracium (Asteraceae) and Their Synthetic and Apomictic Hybrids
Source: Front Plant Sci. 2020 Nov 2;11:591053. doi: 10.3389/fpls.2020.591053 (PMC7667050; doi:10.3389/fpls.2020.591053)
Supplement: Supplementary Figure 1 — Graphical representation of the newly detected satellite repeats. (A) RepeatExplorer graph layouts of the respective clusters showing a circular shape, typical for tandem repeats. (B) Self-similarity dot-plots of the cluster contigs created by the YASS genomic similarity search tool. The tandemly repeated motifs are displayed as green, parallel, diagonal lines, while the distance between lines equals lengths of the motifs. The red lines perpendicular to the main diagonal lines indicate inverted repeats. [file Image_1.pdf]

Supplementary Figure 1 | Graphical representation of the newly detected satellite repeats

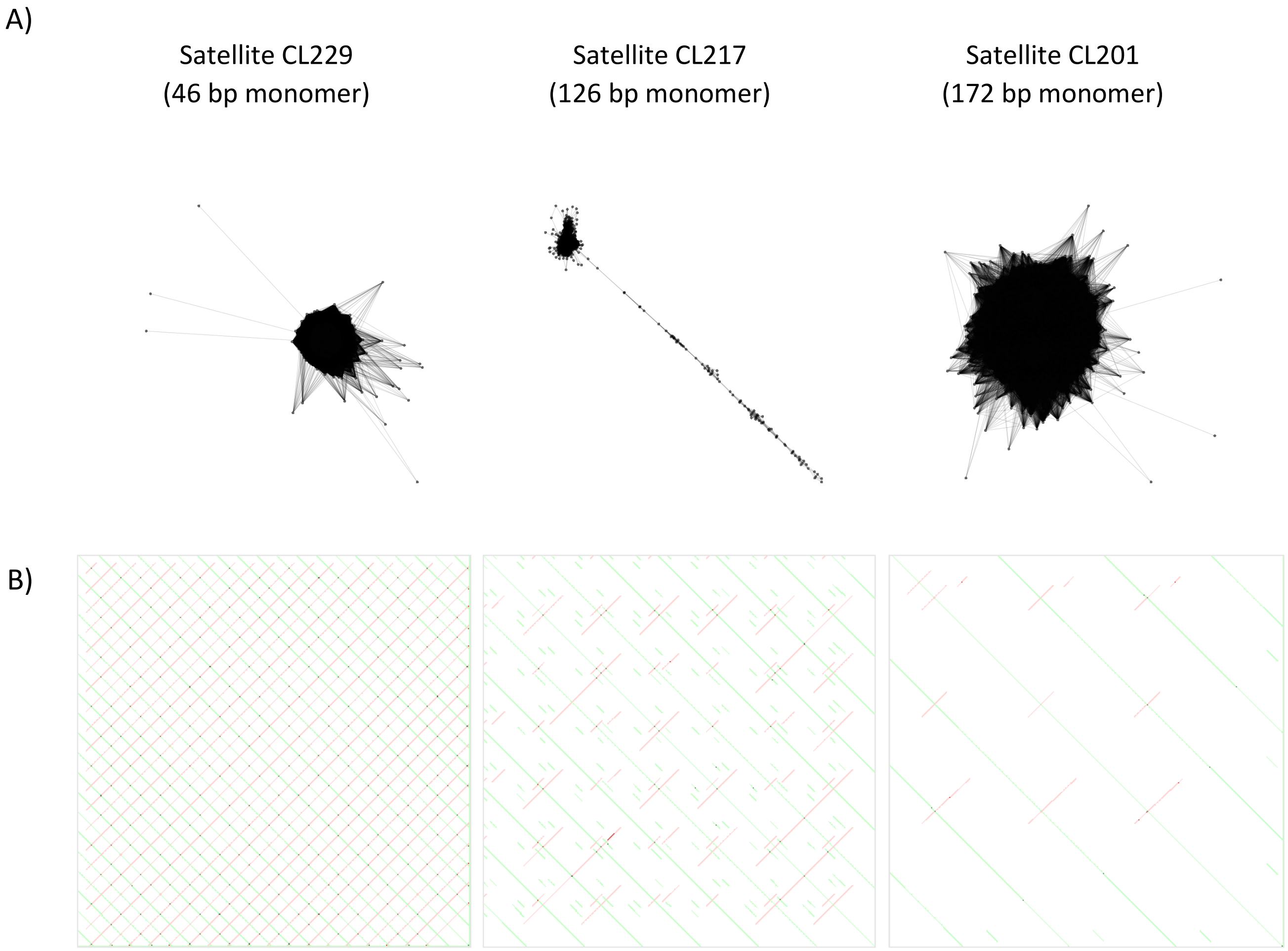

A) RepeatExplorer graph layouts of the respective clusters showing a circular shape, typical for tandem repeats. B) Self-similarity dot-plots of the cluster contigs created by the YASS genomic similarity search tool. The tandemly repeated motifs are displayed as green, parallel, diagonal lines, while the distance between lines equals lengths of the motifs. The red lines perpendicular to the main diagonal lines indicate inverted repeats.
