# Supplementary material for: Characterization and Dynamics of Repeatomes in Closely Related Species of Hieracium (Asteraceae) and Their Synthetic and Apomictic Hybrids
Source: Front Plant Sci. 2020 Nov 2;11:591053. doi: 10.3389/fpls.2020.591053 (PMC7667050; doi:10.3389/fpls.2020.591053)
Supplement: Supplementary Figure 2 — An example of summary histogram of RE clustering analysis in Hieracium (accession IntA). Superclusters are represented as columns in the histogram; the width of the columns corresponds to the size of superclusters. The horizontal lines within columns represent clusters in descending order of their sizes. The largest supercluster (no. 1) contains 23,14% of input reads and the first five superclusters contain 49% of all input reads. [file Image_2.pdf]

Supplementary Figure 2 | An example of summary histogram of RE clustering analysis in *Hieracium* (accession IntA)

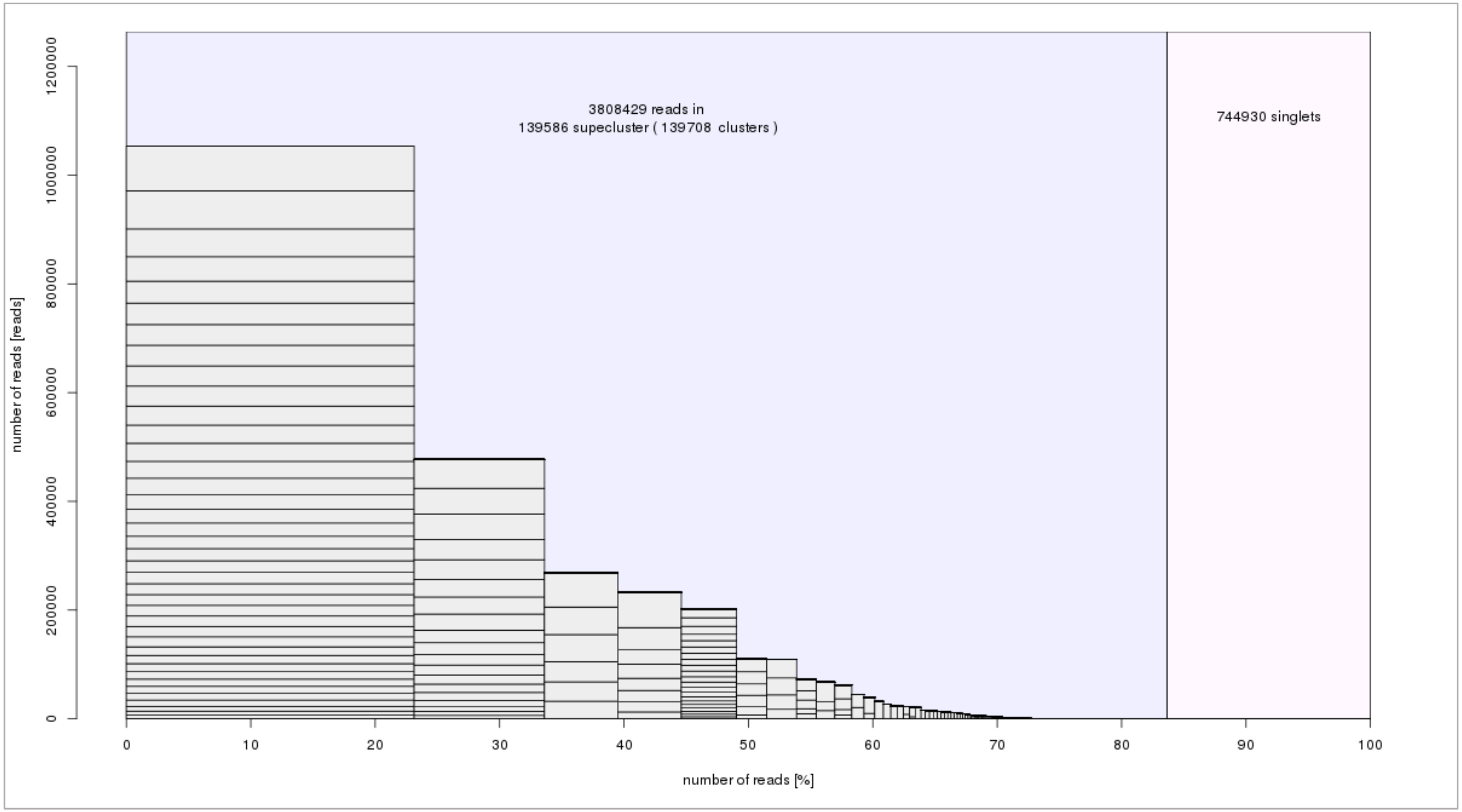

Superclusters are represented as columns in the histogram; the width of the columns corresponds to the size of superclusters. The horizontal lines within columns represent clusters in descending order of their sizes. The largest supercluster (no. 1) contains 23,14% of input reads and the first five superclusters contain 49% of all input reads.
