# Supplementary material for: Characterization and Dynamics of Repeatomes in Closely Related Species of Hieracium (Asteraceae) and Their Synthetic and Apomictic Hybrids
Source: Front Plant Sci. 2020 Nov 2;11:591053. doi: 10.3389/fpls.2020.591053 (PMC7667050; doi:10.3389/fpls.2020.591053)

Supplementary Figure 3 | Comparative analyses of parental species and their natural and synthetic hybrids.
A) to **D)**: Comparative analyses of natural triploid hybrids and representatives of parental species (IntX - created by pooling reads from both individuals of *H. intybaceum*; PreX - created by pooling reads from both individuals of *H. prenanthoides*. **E)** to **G)**: Comparative analyses of synthetic diploid hybrids and their actual parents.
In **A)** to **D),** the numbers of reads of triploids were recalculated using the monoploid genome size (1Cx), in order to make them comparable to homoploid genome size (1C) of diploid parents.

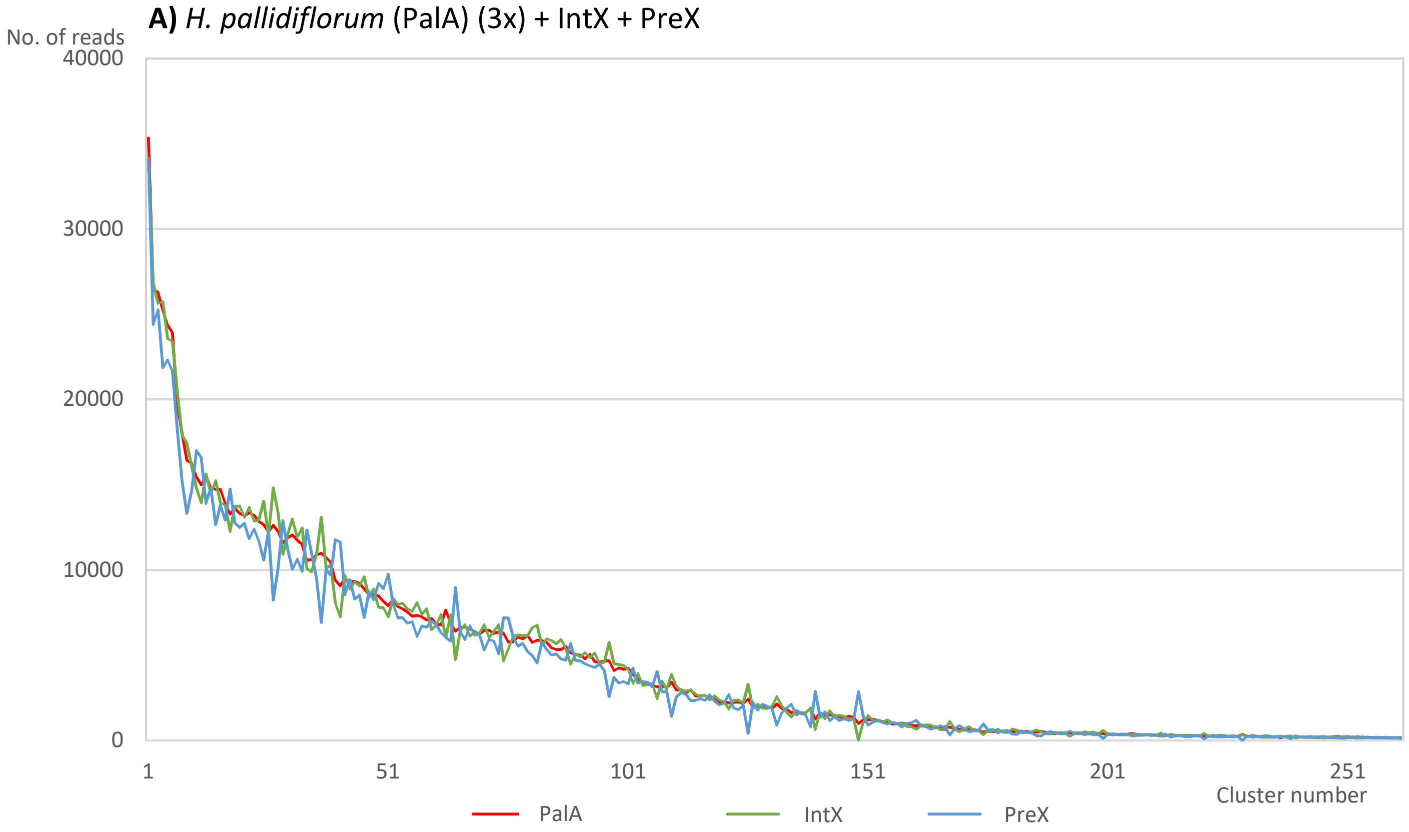

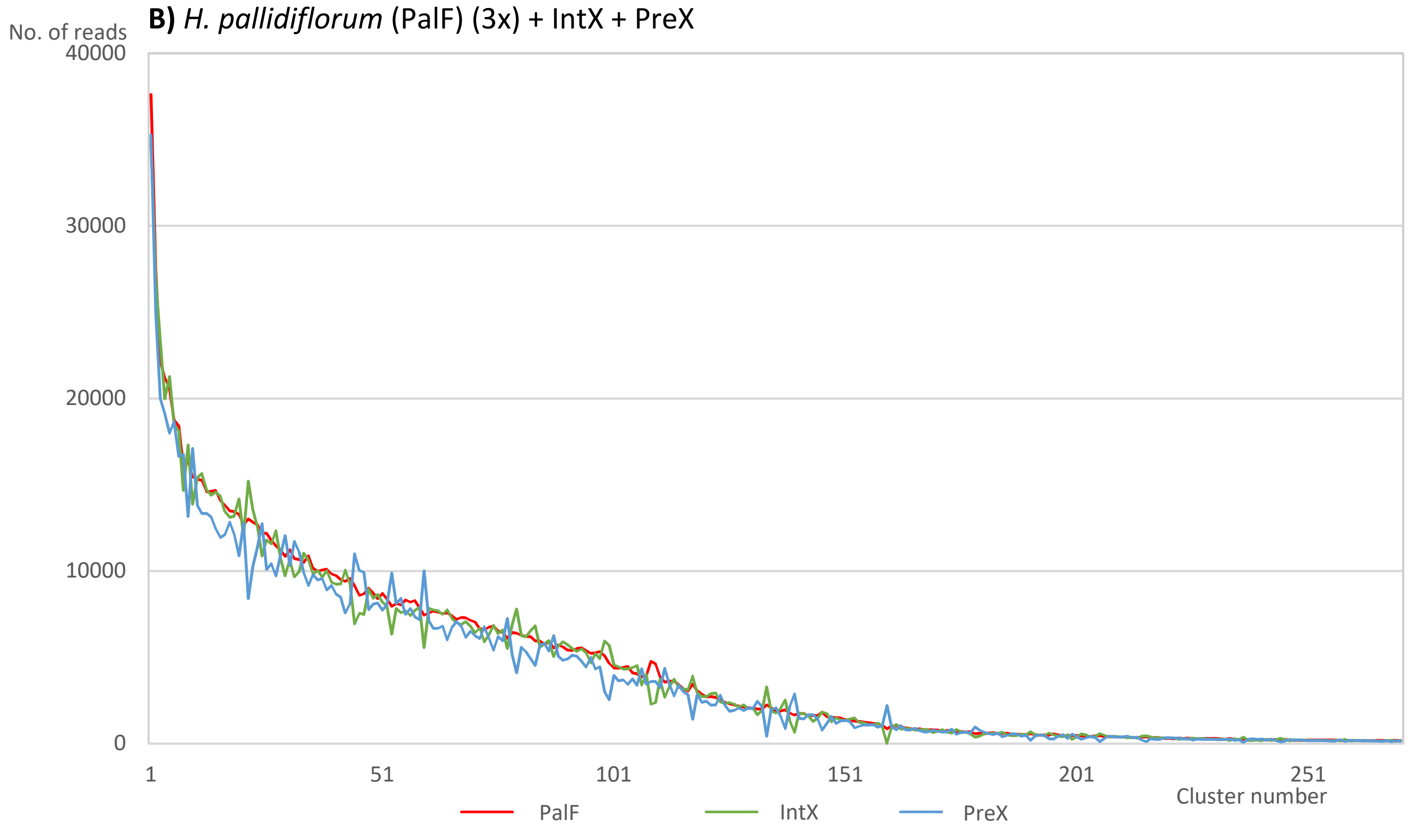

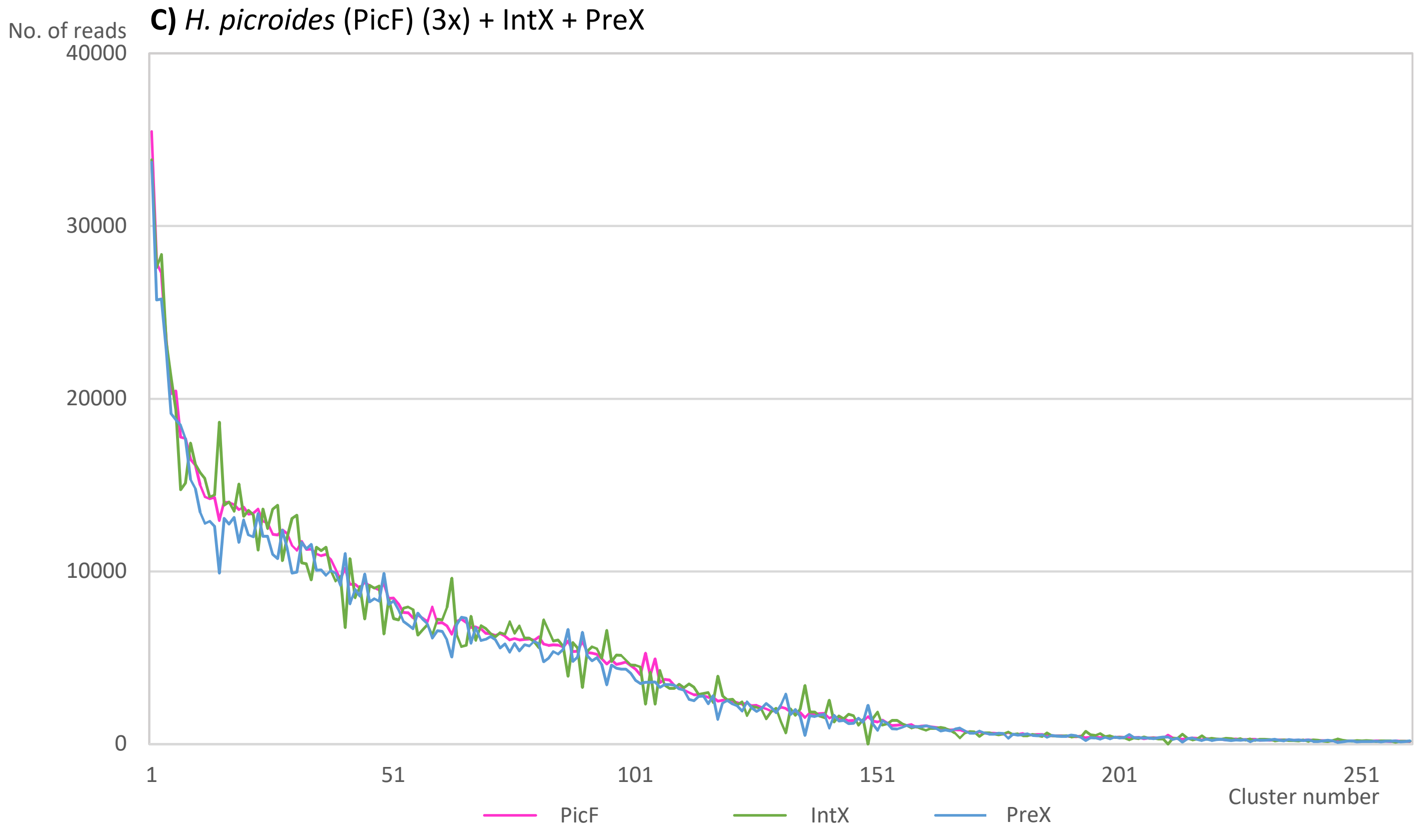
No. of reads
C) H. picroides (PicF) (3x) + IntX + PreX
40000
30000
20000
10000
0
1
51
101
151
201
251
Cluster number
PicF
IntX
PreX

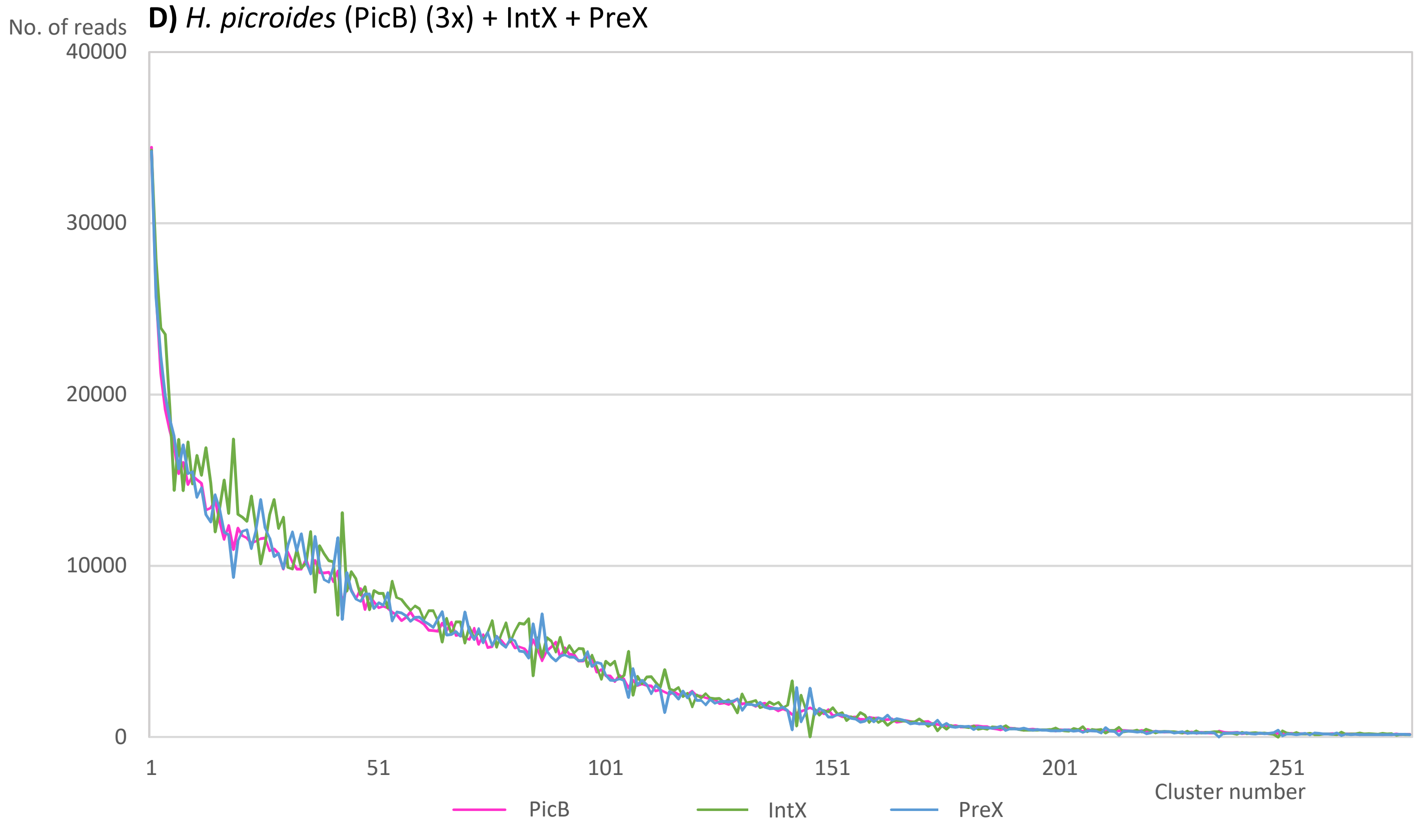
No. of reads
D) H. picroides (PicB) (3x) + IntX + PreX
40000
30000
20000
10000
0
1
51
101
151
201
251
Cluster number
PicB
IntX
PreX

E) Synthetic hybrid (Hyb2) (2x) + *H. intybaceum* (IntA) + *H. prenanthoides* (PreC)

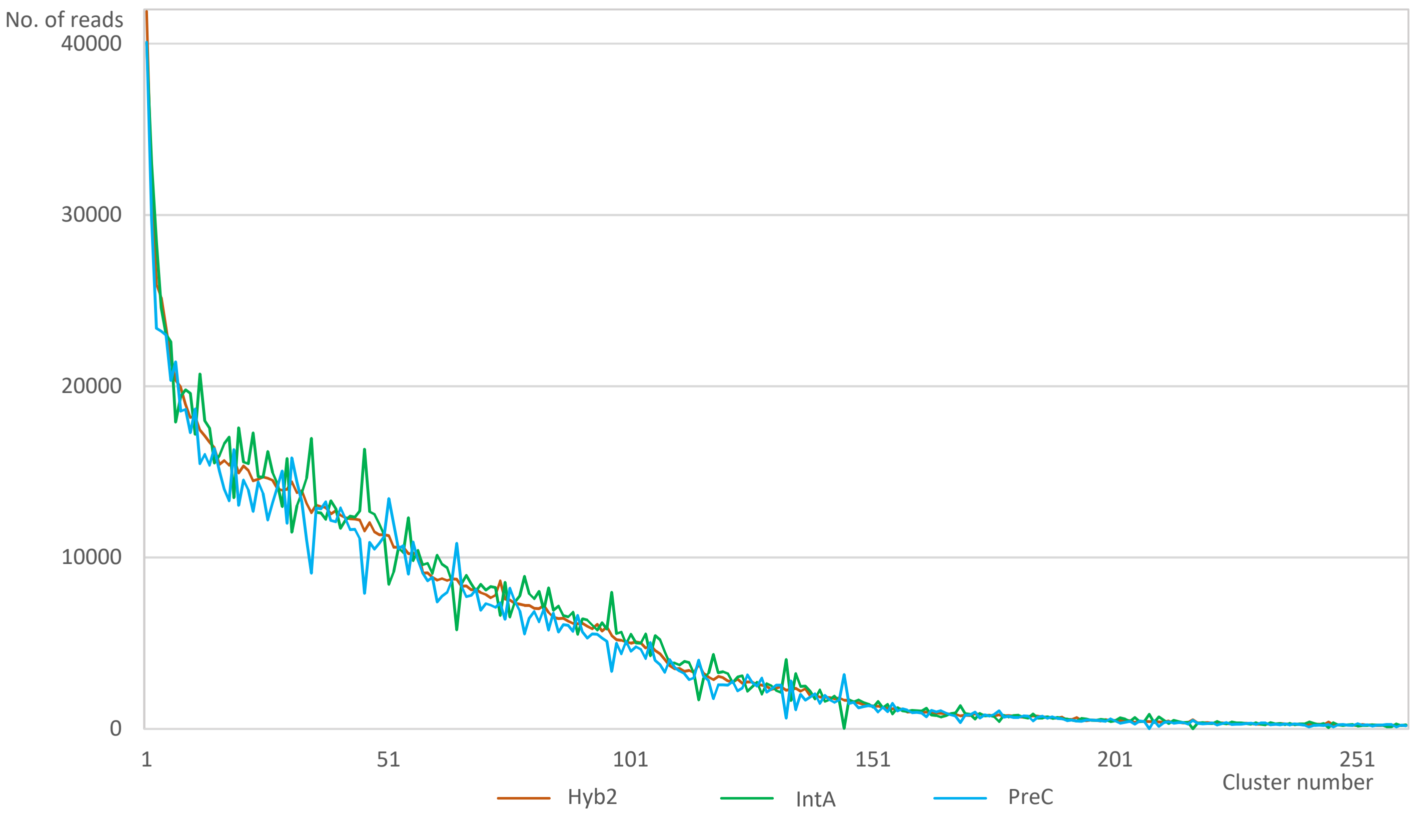

F) Synthetic hybrid (Hyb3) (2x) + *H. intybaceum* (IntA) + *H. prenanthoides* (PreC)

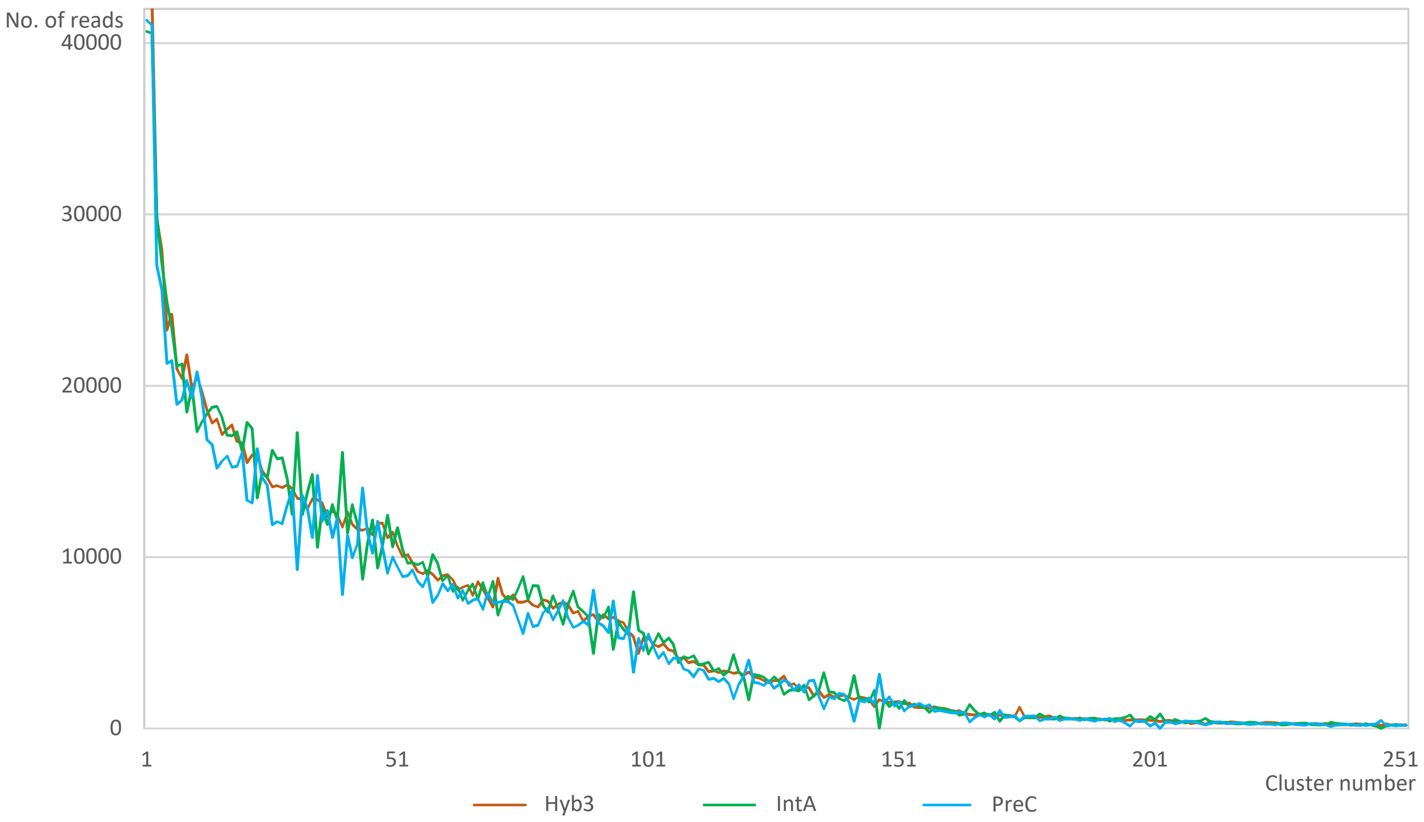

G) Synthetic hybrid (Hyb4) (2x) + *H. intybaceum* (IntA) + *H. prenanthoides* (PreC)

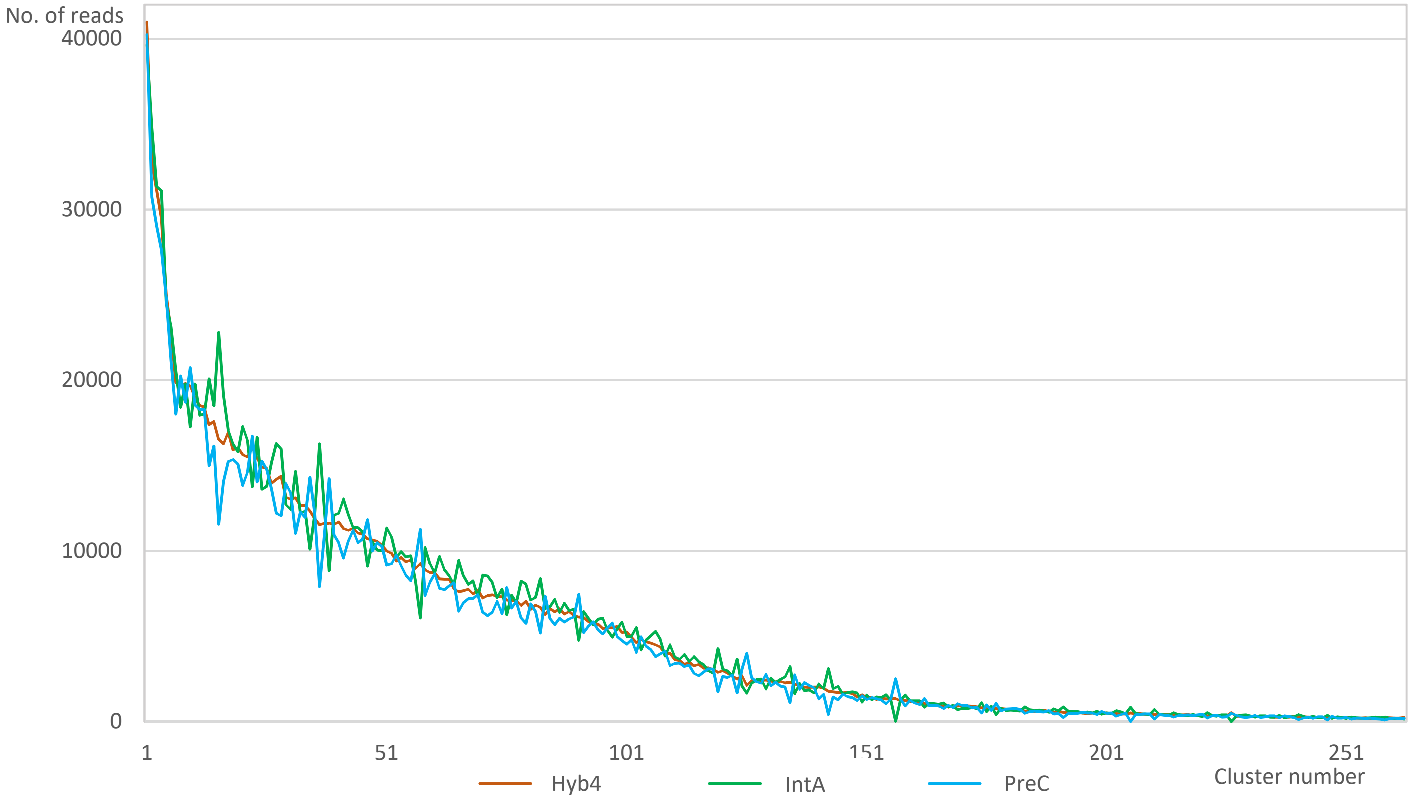

Supplement: Supplementary Figure 3 — Comparative analyses of parental species and their natural and synthetic hybrids. (A–D) Comparative analyses of natural triploid hybrids and representatives of parental species (IntX - created by pooling reads from both individuals of H. intybaceum; PreX - created by pooling reads from both individuals of H. prenanthoides. (E–G) Comparative analyses of synthetic diploid hybrids and their actual parents. In (A–D), the numbers of reads of triploids were recalculated using the monoploid genome size (1Cx), in order to make them comparable to homoploid genome size (1C) of diploid parents. [file Image_3.pdf]
