# Supplementary material for: Characterization and Dynamics of Repeatomes in Closely Related Species of Hieracium (Asteraceae) and Their Synthetic and Apomictic Hybrids
Source: Front Plant Sci. 2020 Nov 2;11:591053. doi: 10.3389/fpls.2020.591053 (PMC7667050; doi:10.3389/fpls.2020.591053)

Supplementary Figure 4 | Cumulative deviation scores of natural and synthetic *Hieracium* hybrids.

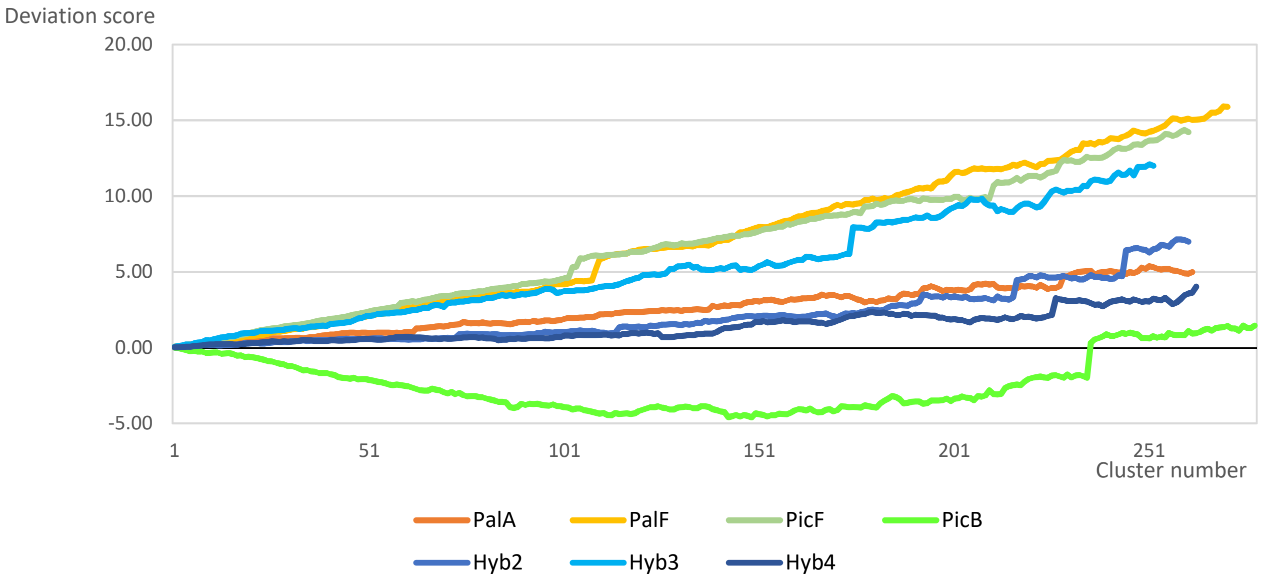

Supplement: Supplementary Figure 4 — Cumulative deviation scores of natural and synthetic Hieracium hybrids. [file Image_4.pdf]
