# Supplementary material for: Characterization and Dynamics of Repeatomes in Closely Related Species of Hieracium (Asteraceae) and Their Synthetic and Apomictic Hybrids
Source: Front Plant Sci. 2020 Nov 2;11:591053. doi: 10.3389/fpls.2020.591053 (PMC7667050; doi:10.3389/fpls.2020.591053)
Supplement: Supplementary Figure 5 — Deviation scores of pararetrovirus clusters. Gray triangles show deviation scores of both pararetrovirus clusters detected in the seven comparative Repeat Explorer analyses. For combinations of samples subjected to comparative analyses, see text and Table 3. Deviation scores were grouped by hybrid origin (boxplots) and compared via ANOVA. The deviation score in pararetrovirus clusters was significantly higher in natural hybrids (mean deviation scores: synthetic hybrids −0.04, natural hybrids 0.26; ANOVA: F = 27.2; p < 0.001). ***p < 0.01; syn – synthetic hybrids; nat – natural hybrids. [file Image_5.pdf]

Supplementary Figure 5 | Deviation scores of pararetrovirus clusters

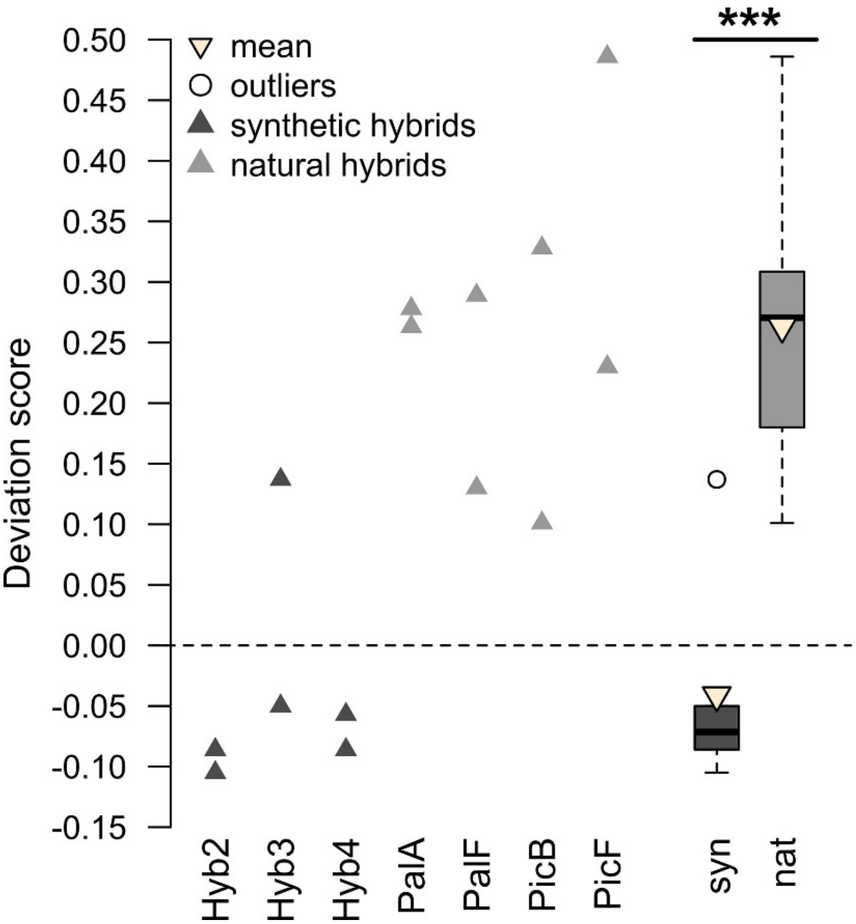

Grey triangles show deviation scores of both pararetrovirus clusters detected in the seven comparative RepeatExplorer analyses. For combinations of samples subjected to comparative analyses, see text and Table 3. Deviation scores were grouped by hybrid origin (boxplots) and compared via ANOVA. The deviation score in pararetrovirus clusters was significantly higher in natural hybrids (mean deviation scores: synthetic hybrids -0.04, natural hybrids 0.26; ANOVA: $F=27.2$; $p < 0.001$). *** – $p < 0.01$; syn – synthetic hybrids; nat – natural hybrids.
