# Supplementary material for: Characterization and Dynamics of Repeatomes in Closely Related Species of Hieracium (Asteraceae) and Their Synthetic and Apomictic Hybrids
Source: Front Plant Sci. 2020 Nov 2;11:591053. doi: 10.3389/fpls.2020.591053 (PMC7667050; doi:10.3389/fpls.2020.591053)
Supplement: Supplementary Figure 6 — Chromosomal localization of CL82 by FISH in synthetic and natural hybrids. (A–C) Synthetic F1 diploid hybrids. (D–G) Natural triploid hybrids. FISH signals: CL82 (purple signal, magenta arrows), 45S rDNA (green signal), 5S rDNA (red signal). Chromosomes were counterstained with DAPI (blue). Bars = 5 μm. [file Image_6.pdf]

Supplementary Figure 6 | Chromosomal localization of CL82 by FISH in synthetic and natural hybrids

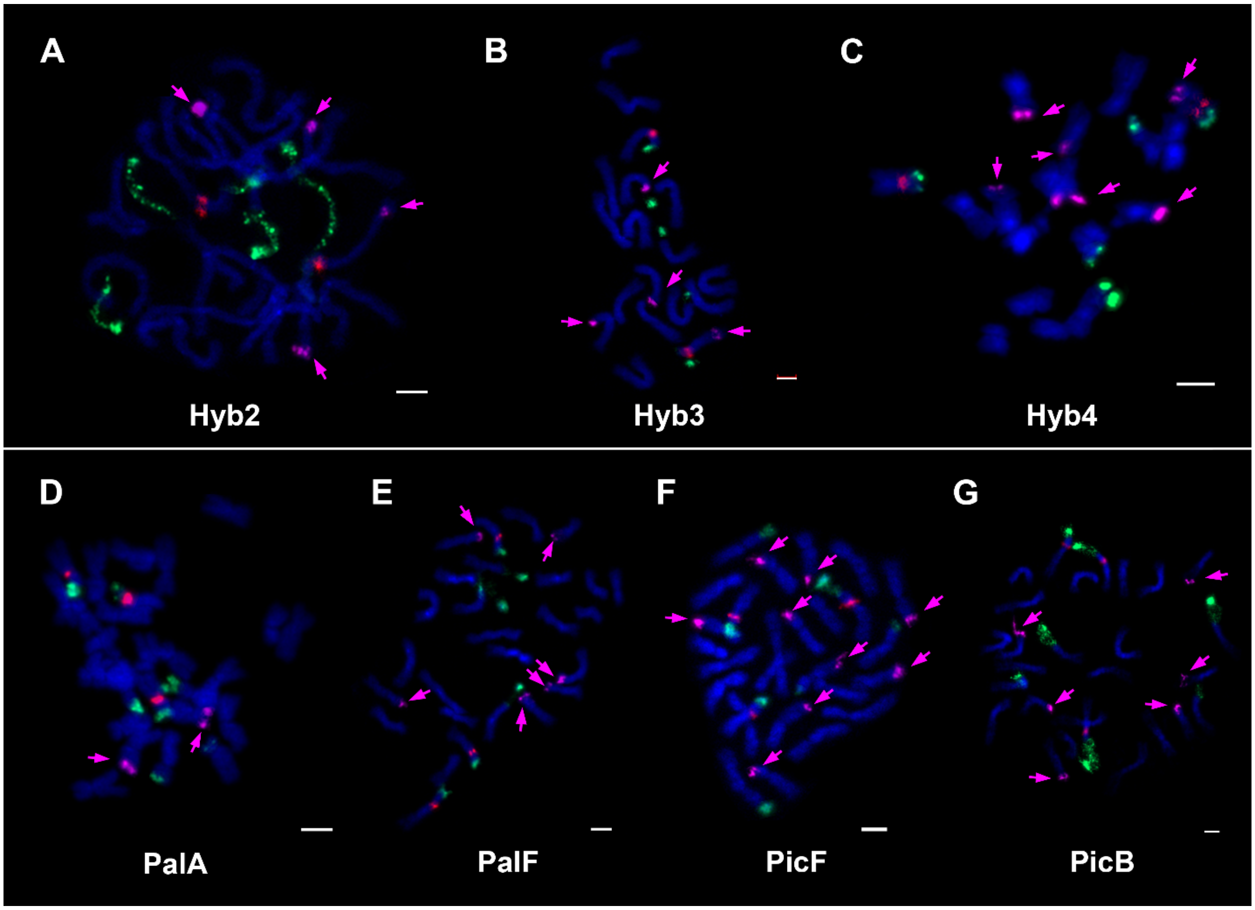

(A–C) Synthetic F1 diploid hybrids. (D–G) Natural triploid hybrids. FISH signals: CL82 (purple signal, magenta arrows), 45S rDNA (green signal), 5S rDNA (red signal). Chromosomes were counterstained with DAPI (blue). Bars = 5 µm.
